# *Informational structures*: A dynamical system approach for integrated information

**DOI:** 10.1371/journal.pcbi.1006154

**Published:** 2018-09-13

**Authors:** Francisco J. Esteban, Javier A. Galadí, José A. Langa, José R. Portillo, Fernando Soler-Toscano

**Affiliations:** 1 Department of Experimental Biology, University of Jaén, Jaén, Spain; 2 Department of Differential Equations and Numerical Analysis, University of Seville, Seville, Spain; 3 Department of Applied Mathematic I, University of Seville, Seville, Spain; 4 Department of Philosophy, Logic and Philosophy of Science, University of Seville, Seville, Spain; Institut de Neurosciences des Systèmes, FRANCE

## Abstract

Integrated Information Theory (IIT) has become nowadays the most sensible general theory of consciousness. In addition to very important statements, it opens the door for an abstract (mathematical) formulation of the theory. Given a mechanism in a particular state, IIT identifies a conscious experience with a conceptual structure, an informational object which exists, is composed of identified parts, is informative, integrated and maximally irreducible. This paper introduces a space-time continuous version of the concept of integrated information. To this aim, a graph and a dynamical systems treatment is used to define, for a given mechanism in a state for which a dynamics is settled, an *Informational Structure*, which is associated to the global attractor at each time of the system. By definition, the informational structure determines all the past and future behavior of the system, possesses an informational nature and, moreover, enriches all the points of the phase space with cause-effect power by means of its associated Informational Field. A detailed description of its inner structure by invariants and connections between them allows to associate a transition probability matrix to each informational structure and to develop a measure for the level of integrated information of the system.

## Author summary

In this paper we introduce a space-time continuous version for the level of integrated information of a network on which a dynamics is defined. The concept of integrated information comes from the IIT of consciousness. By a strict mathematical formulation, we complement the existing IIT theoretical framework from a dynamical systems perspective. In other words, we develop the bases for a continuous mathematical approach to IIT introducing a dynamical system as the driving rule of a given mechanism. We also introduce and define the concepts of *Informational Structure* and *Informational Field* as the complex network with the power to ascertain the dynamics (past and future scenarios) of the studied phenomena. The detailed description of an informational structure is showing the cause-effect power of a mechanism in a state and thus, a characterization of the quantity and quality of information, and the way this is integrated. We firstly introduce how network patterns arise from dynamic phenomena on networks, leading to the concept of informational structure. Then, we formally introduce the mathematical objects supporting the theory, from graphs to informational structures, throughout the integration of dynamics on graphs with a global model of differential equations. After this, we formally present some of the IIT’s postulates associated to a given mechanism. Finally, we provide the quantitative and qualitative characterization of the integrated information, and how it depends on the geometry of the mechanism.

## Introduction

Dynamical Systems and Graph Theory are naturally coupled since any real phenomenon is usually described as a complex graph in which the evolution of time produces changes in specific measures on nodes or links among them [1, 2]. In this work, the starting point is any structural network, including a parcelling of the brain, possessing an intrinsic dynamics. For brain dynamics, the collective behavior of a group of neurons can be represented as a node with a particular dynamics along time [3–6]. In general, the mathematical way to describe and characterize dynamics is by (ordinary or partial) differential equations (continuous time) [7] or difference equations (discrete time) [8]. Global models on brain dynamics are grounded on anatomical structural networks built under parcelling of the brain surface [9–11]. Indeed, they are based on systems of differential equations described on complex networks, which may include noise, delays, and time-dependent coefficients. Thus, the designed dynamical system models the activity of nodes connected to each other by a given adjacency matrix. A global dynamics emerges through simulated dynamics at each node, which is coupled to others as detailed in the anatomical structural network (see, for instance, [12] for the structural networks on primate connectivity). Then, an empirical functional network and a simulated functional network emerge by correlation or synchronization of data on the structural network [4, 13, 14], showing a similar behavior and topology after a proper fitting of the parameters in the differential equations associated to the dynamics.

We take advantage of this approach to apply some of the main results on the modern theory of dynamical systems showing that, given a dynamics on a network, there exists an object, the global attractor [15–18], determining all the asymptotic behaviour of each state of the network. The attractor exists and its nature is essentially informational, as it possesses the power to produce a curvature of the phase space enriching every point with the information on its possible past and future dynamics. The structure of the global attractors (or attracting complex networks [19, 20]), described as composed structures by invariants and connections, naturally shows that its information is structured, composed by different parts, and can be unreachable from the study of the information of its parts, so allowing for a definition of integrated information.

Integrated Information Theory (IIT, [21]), created by G. Tononi [22–24] starts with a phenomenological approach to the theory of consciousness. It assumes that consciousness exists and tries to describe it by defining the axioms that it satisfies. Having the axioms on hand, they serve to introduce the postulates that every physical mechanism has to obey in order to produce a conscious experience. This fact opens the door to the possibility of the mathematization of the theory by defining and describing postulates on concrete networks where a dynamics can be settled. It is then possible to define the appropriate structured dynamics which is supposed to explain a conscious experience by preserving its axioms. The IIT approach allows to represent a conscious experience and even to measure it quantitatively and qualitatively by the so called integrated information Φ^*max*^, which, at the same time indicates that, at the base of consciousness, there are essentially phenomena of causal processes of integrated information nature [25]. This fact links IIT to Information Theory and the Theory of Causality [26]. On the mathematical level, IIT approach is based on graphs consisting of logic gates and transition probabilities describing causality of consecutive discrete states on those graphs [21].

**Fig 1:**
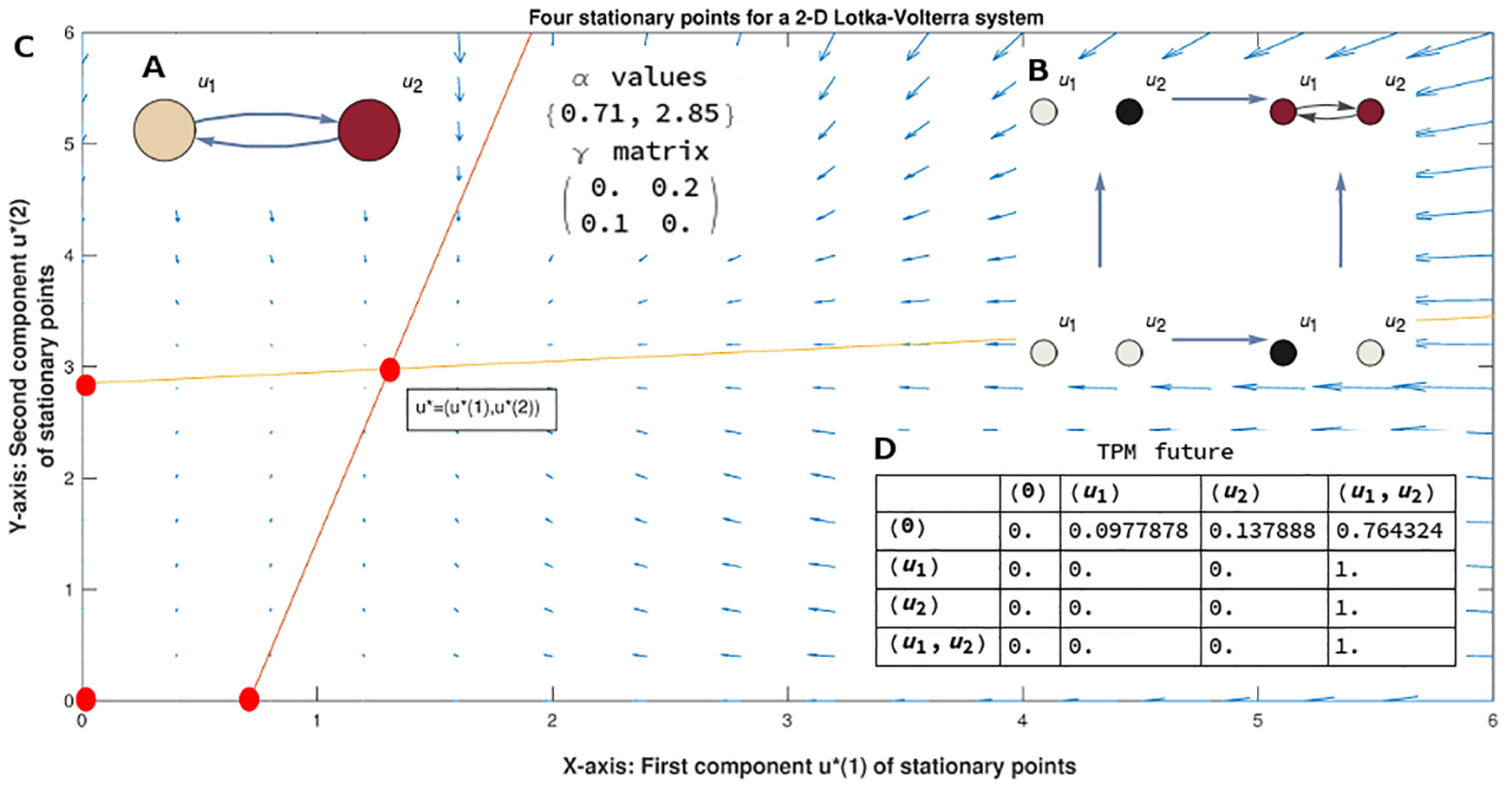
**A**. A mechanism (graph) of two nodes where a system of two differential equations given by (7) is defined, one for each node, using the given values for the *α* and *γ* parameters. **B**. The Informational Structure (IS) is a new complex network made by four stationary points (equilibria) and directed links defined by global solutions. Each stationary point of the IS is a state associated to a subgraph of the original mechanism (non-null existing nodes are shown in black). The actual state of the mechanism corresponds to a state of the IS, highlighted in pink at the figure (the state where both *u*_1_ and *u*_2_ have a value greater than 0). **C**. (background) The associated directional field describing the tangent directions of trajectories inside the IS. The two straight lines (in orange and yellow) are the nullclines associated to the system and they intersect in three stationary points (except (0, 0)): one is a semitrivial stationary point in the *X* axis; the second is a semitrivial stationary point in the *Y* axis. The last one is the stationary point with two strictly positive values. All of the stationary points constitute the nodes of the IS. Each stationary point is hyperbolic and locally creates a field of directions towards (stability) or from them (unstabilities). The informational field can be globally described by the sum of the stability and unstability influences of each continuous stationary solution passing when going to one node to other in the IS. **D**. The measurement of the amount of information to link any pair of nodes allows to define a Transition Probability Matrix (TPM) with the probability for each state of going to any other. States of the IS are denoted by the list of nodes having a value greater than 0. State (0) represents the node of the IS with both *u*_1_ and *u*_2_ equal to 0.

In this paper we present a continuous-time version (see Section Results for a formal description) related to IIT based on the theory of dynamical systems. IIT bases any particular experience on a mechanism, defined in a particular state, which possesses a well defined cause-effect power. Our starting point is given by a graph describing a mechanism and, thus, a graph is first defined. However, we focus our study on the network patterns arising from dynamical phenomena. So, a dynamical system and the associated mathematical objects (global attractor, equilibrium points, unstable invariant sets) have to be also defined. As a novelty in dynamical systems theory, the global attractor (which, for the gradient case consists of the equilibria and the heteroclinic connections between them [15, 17, 27]) is redefined as an object of informational nature, an *Informational Structure* (IS). An IS is a flow-invariant object of the phase space described by a set of selected invariant global solutions of the associated dynamical system, such as stationary points (equilibria) and connecting orbits among them (Fig 1). This set of invariants inside the IS creates a new structure, a new complex network with the power to ascertain the dynamics (past and future scenarios) of natural phenomena. Every IS posseses an associated *Informational Field* (IF), globally described from the attraction and repulsion rates on the nodes of the IS. We are able to translate the energy landscape caused by the IS and the IF into a transition probability matrix (TPM) to pass from one state to another within the system (see Section Results). Thus, the level of information of a mechanism in a state is going to be given by the global amount of deformation of the phase space caused by the intrinsic power of the IS and IF. The geometrical characterization of ISs can provide both the quality of the related information and, in particular, the shape in which it is integrated in the whole system, allowing to measure the level of integrated information it contains. Thus, the quality of the information comes from the detailed study of informational structure, which now possesses an intrinsic dynamics and enjoys a continuous change. This structure depends on the parameters of the underlying equations and has the ability to possibly rapidly change in the response to the change of those parameters (see Section Materials and methods). From this continuous approach, we are able to introduce first definitions for postulates of existence, composition, information and integration for a mechanism in a state. There is still a gap to the more elaborated formal definitions from IIT 3.0 [21] (see Section Discussion), including the composition and exclusion postulates. However, our framework naturally leads to a study on the continuous dependence between the topology of the network and the level of integrated information for a given mechanism (see Section Results).

## Materials and methods

### Dynamics on graphs

Many real phenomena can be described by a set of key nodes and their associated connections, building a (generically) complex network. In this way, we can always construct an application between a real situation and an abstract graph describing its essential skeleton. An undirected graph is an ordered pair G=(V,E) comprising a non-empty set *V* of vertices (or nodes) together with a set *E* of edges joining 2-element subsets of *V*. The order of a graph is given by the number of nodes, and its size by the number of edges. A directed graph or digraph is a graph in which edges (named arcs) have orientations.

We want to study the behaviour on networks of systems of evolutionary differential equations as

$$\begin{array}{c} \frac{du}{dt}=F\left(t,u\right), \end{array}$$ (1)

where *F* is a nonlinear map from $\left(t,u\right)\in R\times R^{N}$ to $R^{N}$. For modeling purposes, we could also add, for instance, delays, stochastic terms, or to make solution *u*(*t*) also depend on a subset *Ω* of the three dimensional space, i.e. *u*(*t*, *x*), for $x\in \Omega \subset R^{3}.$ Given an initial condition, suppose existence and uniqueness of solutions. If not, a multivalued approach could also be adapted.

The phase space *X* (in our case $X=R^{N}$) represents the framework in which the dynamics described by a group of transformations *S*(*t*): *X* → *X* is developed. Given a *phase space* *X* we define a dynamical system on *X* as a family of non-linear operators ${\left\{S\left(t\right)\right\}}_{t\in R^{+}}$,

$$\begin{array}{c} S(t):X\to X\\ u\in X,\;S(t)u\in X \end{array}$$

which describes the dynamics of each element *u* ∈ *X*. In particular, *S*(*t*)*u*_0_ = *u*(*t*;*u*_0_) is the solution of the differential Eq (1) at time *t* with initial condition *u*_0_.

The global attractor is the central concept in dynamical system theory, since it describes all the future scenarios of a dynamical system. It is defined as follows [15–18, 28, 29]:

A set A⊆X is a global attractor for {*S*(*t*): *t* ≥ 0} if it is

(i) compact,
(ii) invariant under {*S*(*t*): *t* ≥ 0}, i.e. S(t)A=A for all *t* ≥ 0, and
(iii) attracts bounded subsets of *X* under {*S*(*t*): *t* ≥ 0} for the Hausdorff semidistance; that is, for all *B* ⊂ *X* bounded
  $$\begin{array}{c} \lim_{t\to +\infty }{\text{dist}}_{H}\left(S\left(t\right)B,A\right):=\lim_{t\to +\infty }\underset{b\in B}{\sup}\underset{a\in A}{\inf}\left(S\left(t\right)b,a\right)=0. \end{array}$$

Observe that (ii) is showing a crucial property of an attractor, as supposes a set with a proper intrinsic dynamics. Moreover, (iii) points that this set is determining all the future dynamics on the phase space *X*. We say that u* ∈ *X* is an equilibrium point (or stationary solution) for the semigroup *S*(*t*) if *S*(*t*)u* = u*, for all *t* ≥ 0. A stationary point is a trivial case for a global solution associated to *S*(*t*), i.e., ξ:R→X such that *ξ*(*t* + *s*) = *S*(*t*)*ξ*(*s*) for all s∈R, $t\in R^{+}$. Stationary points are the minimal invariant objects inside a global attractor. Every invariant set is a subset of the global attractor [15]. Generically, connections among invariant sets in the attractor describe its structure [27, 30]. To this aim we need the following definitions, which also allow us to define the behaviour *towards the past* in a global attractor.

The unstable set of an invariant set Ξ is defined by

$$\begin{array}{c} W^{\text{u}}\left(\Xi \right)=\{z\in X:\;\text{there}\;\text{is}\;\text{a}\;\text{global}\;\text{solution}\;\xi :R\to X\;\text{for}\;S\left(t\right)\\ \text{satisfying}\;\xi (0)=z{\;\text{and}\;\text{such}\;\text{that}\;\text{lim}}_{t\to -\infty }\text{dist}(\xi (t),\Xi )=0\}. \end{array}$$

The stable set of an invariant set Ξ is defined by

$$\begin{array}{c} W^{s}\left(\Xi \right)=\left\{z\in X:\;\text{such}\;\text{that}\;\lim_{t\to +\infty }\text{dist}\left(S\left(t\right)z,\Xi \right)=0\right\}. \end{array}$$

We have to think in a global attractor as a set which does not depend on initial conditions, with an intrinsic proper dynamics, composed by a set of special solutions (global solutions), which are connecting particular invariants, so generating a complex directed graph. Moreover, the global attractor has the following properties [17, 18]:

1. It is the maximal invariant set in the phase space.
2. It is the smallest closed attracting set.
3. It is made of bounded complete solutions, i.e., solutions that exists for all time t∈R, and so giving information for the asymptotic past of the system.
4. Generically, its structure is described by invariant subsets and connecting global solutions among them [31, 32].

### The *Fundamental Theorem of Dynamical Systems*

The *Fundamental Theorem of Dynamical Systems* [33] states that every dynamical system on a compact metric space *X* (the one defined on a global attractor, for instance) has a geometrical structure described by a (finite or countable) number (indexed by *I*) of sets {*E*_*i*_}_*i*∈*I*_ with an intrinsic recurrent dynamics and a gradient-like dynamics outside them. In other words, when we define a dynamical system on a graph, the attractor can be always described by a (finite or countable) number of invariants and connections between them.

#### Gradient attractors

We say [15, 17, 31] that a semigroup {*S*(*t*): *t* ≥ 0} with a global attractor A and a disjoint family of isolated invariant sets **E** = {*E*_1_, ⋯, *E_n_*} is a *gradient semigroup* with respect to **E** if there exists a continuous function V:X→R such that

(i) [0,∞)∍t↦V(S(t)u)∈R is non-increasing for each *u* ∈ *X*
(ii) *V* is constant in *E*_*i*_, for each 1 ≤ *i* ≤ *n*; and
(iii) *V*(*S*(*t*)*u*) = *V*(*u*) for all *t* ≥ 0 if and only if $u\in \overset{n}{\underset{j=1}{\cup }}E_{i}.$

In this case we call *V* a Lyapunov functional related to **E**. For gradient semigroups, the structure of the global attractor can be described as follows [15, 27]: Let {*S*(*t*): *t* ≥ 0} be a gradient semigroup with respect to the finite set **E** ≔ {*E*_1_, *E*_2_, ⋯, *E_n_*}. If {*S*(*t*): *t* ≥ 0} has a global attractor A, then A can be written as the union of the unstable manifolds related to each set in **E**, i.e,

$$\begin{array}{c} A=\overset{n}{\underset{j=1}{\cup }}W^{u}(E_{j}). \end{array}$$ (2)

When *E*_*j*_ are equilibria $u_{j}^{*}$, the attractor is described as the union of the unstable manifolds associated to them

$$\begin{array}{c} A=\overset{n}{\underset{j=1}{\cup }}W^{u}(u_{j}^{*}). \end{array}$$

This description of a gradient system shows a geometrical picture of the global attractor, in which all the stationary points or isolated invariant sets (also defined as *Morse sets*, [31, 32]) are ordered by connections related to its *level of attraction* [34] or stability. They conform a Morse decomposition of the global attractor [32, 33, 35–37].

When we refer to the global attractor for the dynamics on a graph, observe that each node given by a partially feasible equilibrium point in the attractor represents an attracting complex subgraph of the original one. Thus, the attractor can be understood as *a new complex dynamical network describing all the possible feasible future networks* [19, 20]. In particular, it contains all the information related to future scenarios of the model.

#### Energy levels

Any Morse decomposition **E** = {*E*_1_, ⋯, *E*_*n*_} of a compact invariant set A leads to a partial order among the isolated invariant sets *E*_*i*_; that is, we can define an order between two isolated invariant sets *E*_*i*_ and *E*_*j*_ if there is a chain of global solutions

$$\begin{array}{c} \left\{\xi_{\ell },\;1\leq \ell \leq r\right\} \end{array}$$ (3)

with

$$\underset{t\to -\infty }{\lim}\text{dist}(\xi_{l}\left(t\right),E_{l})=0$$

and

$$\underset{t\to \infty }{\lim}\text{dist}(\xi_{l}\left(t\right),E_{l+1})=0$$

1 ≤ *ℓ* ≤ *r* − 1, with *E*_1_ = *E*_*i*_ and *E*_*r*_ = *E*_*j*_.

This implies that, given any dynamically gradient semigroup with respect to the disjoint family of isolated invariant sets **E** = {*E*_1_, ⋯, *E*_*n*_}, there exists a partial order in **E**. In [34] (see also [38]) it is shown that there exists a Morse decomposition given by the so-called energy levels $N=\{N_{1},N_{2},\cdots ,N_{p}\}$, *p* ≤ *n*. Each of the levels $N_{i}$, 1 ≤ *i* ≤ *p* is made of a finite union of the isolated invariant sets in **E** and **N** is totally ordered by the dynamics defined by (3). Indeed, the associated Lyapunov function has strictly decreasing values in any two different level-sets of **N** and any two elements of **E** which are contained in the same element of **N** (same energy level) are not connected.

#### Attracting invariant networks

The existence of a global attractor implies that when we look at the evolution in time of each *u*_*i*_(*t*) we can see the “picture” in asymptotic time drawn by all $\left(u_{1}\left(t\right),u_{2}\left(t\right),\ldots ,u_{N}\left(t\right)\right)\in R^{N}$. Thus, this evolution is engaged with an invariant attracting network in which

i) Each node represents a stationary solution (or, generically, a minimal invariant subset of the global attractor), which can be described as a binary vector representing the activation or inactivation of nodes, i.e., its points constitute a subgraph of the former graph representing the modelled system.
ii) There exist oriented connections between the above subgraphs, so leading to a directed graph.
iii) Each subgraph has totally determined stability properties; i.e., it is known not only which other nodes are connected to it or to which it is connected, but also, for instance, the velocity or the tendency for the attraction.
iv) The whole invariant structure is determining the long time behaviour of all solutions of the system.

In summary, *behind* a graph with dynamics (a mechanism in our conception) there exists a new complex network of connected subgraphs, governing all possible scenarios of the system.

### Informational structures: A formal definition

The starting point in our approach is a system of connected elements where a dynamics is defined. This system is called a mechanism. Composition and exclusion postulates in IIT 3.0 allow to consider mechanisms given by any subset of the system. Here, we are only focusing in the mechanism given by the whole system. A global attracting network can be characterized by the amount of information it provides to the mechanism, since the nature of a global attractor is essentially informational. Indeed, the informational nature of the attractor is based on the following assertions:

1. They live in the phase space *X*, an abstract formulation for the description of the flow.
2. Their existence is not experimentally established, i.e., they are not necessarily related to physical experiments. They exist, associated and grounded to a particular mechanism, as a (small) compendium of “selected solutions”, which forms a complex structure and, moreover, determines the behaviour of all other solutions.

The state of a mechanism is given by the state of its nodes. If those nodes take real values, the state is given by a vector of real numbers. When a dynamical system is defined for a mechanism in a state, it has cause-effect power, meaning that it conditions the possible past and future states of the mechanism. We can find the cause-effect power of a mechanism in a state by looking at its (local) attracting invariant sets. Typically, these are stationary points or periodic orbits [32, 39–41], but it could also contain invariant sets with chaotic dynamics [42–44]. Following IIT, we will measure the amount of information on the structure made by these invariants, in the sense of its power to restrict the past and the future states. In IIT language, this is *intrinsic information*: differences (state of a mechanism) that make a difference (restricted set of possible past and future states) within a system (see the IIT glossary in [21]).

Now we can define an Informational Structure (IS). Suppose we have a complex graph G given by *N* nodes and links among them. We denote by *G*_*i*_ every subset of G. An informational structure is a complex graph $I=\{G_{1},\cdots ,G_{M}\}$ which nodes are subgraphs *G*_*i*_ of G and links among them, with the following properties

1. Links between nodes are directed by their dynamics.
2. Each link has a defined weight.
3. Each link has a dynamical behaviour given by a model of differential equations.
4. Dynamics are defined by stability of nodes.

**Fig 2:**
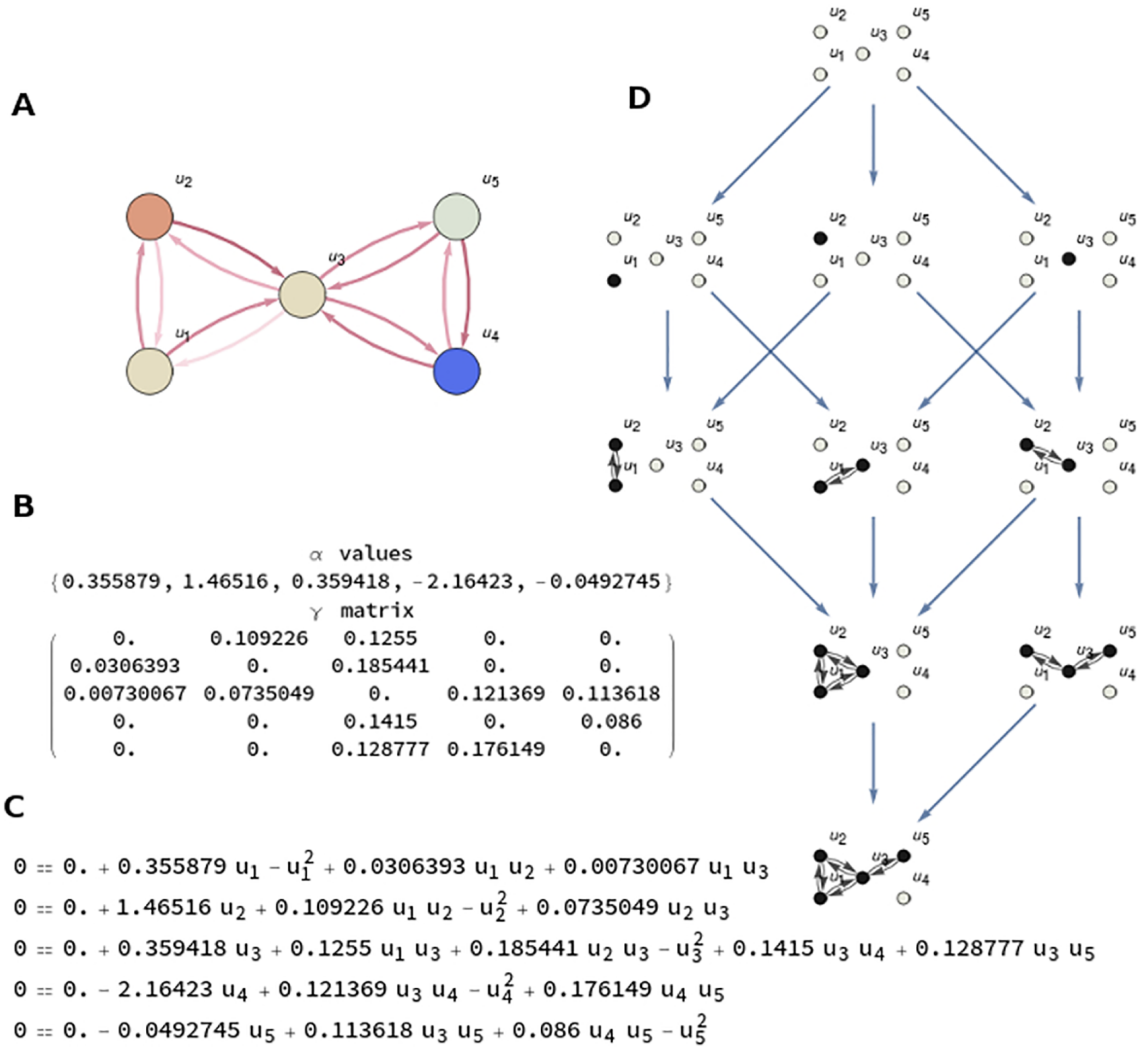
**A**. Mechanism with five nodes *u*_1_, ⋯, *u*_5_ representing five interacting components represented as a graph G. **B**. *α* values and *γ* values (position (*i*, *j*) in the matrix represents the influence of node *u*_*i*_ over *u*_*j*_) are the parameters defining the dynamics of the system. **C**. Nonlinear system of five equations to calculate the stationary points of the dynamical system. **D**. Informational Structure (IS) associated to the mechanism. Observe that the IS is a new complex network made by directed links, related to dynamics, of subgraphs *G*_*i*_ of the original one G. Each node in the IS contains a stationary point of the dynamical system. Nodes of each *G*_*i*_ with a value greater than 0 are shown in black. Grey nodes have value 0. Arrows in the IS relates to the cause-effect power of each state, going from one state to another. The relation induced by the arcs is transitive, but only the minimal arcs to understand the dynamics of the IS are represented.

An IS (see Fig 2) becomes a natural emerging object (of informational nature) from a given mechanism in a state with intrinsic cause-effect power to the past and the future. Moreover, some properties of ISs can be given:

1. ISs exist and are composed by a set of simple elements. They have cause-effect power, producing intrinsic information within the mechanism.
2. At any instant, an IS determines all the subset of past and futures states.
3. The quantity of information can be measured, for any given time, by the size of the IS at time *t*. Quality of the information is related to the shape of the IS.
4. Integration can be measured by partitions of the global graph.

#### A global model

Lotka-Volterra models have been used to generate reproducible transient sequences in neural circuits [45–49]. In our case, for a general model for *N* nodes, we define a system of *N* differential equations given by:

$$\begin{array}{c} \frac{du_{i}}{dt}=u_{i}(\alpha_{i}+\sum_{j=1}^{N}\gamma_{ij}u_{j}),\;\;\;\;i=1,...,N, \end{array}$$ (4)

where the matrix Γ = (*γ*_*ij*_) ∈ *R*^*N*×*N*^ is referred to the interaction-matrix. In matrix formulation, (4) reads as

$$\begin{array}{c} \frac{du}{dt}=u(\alpha +\Gamma u),\;\;\;\; \end{array}$$ (5)

with $A\in R^{N^{2}}$ and $\alpha \in R^{N}$. Given an initial data for (5), sufficient conditions for the existence and uniqueness of global solutions are well-known (see, for instance, [50, 51]) The phase space for (5) is the positive orthant

$$\begin{array}{c} R_{+}^{N}=\left\{u=\left(u_{1},\cdots ,u_{N}\right)\in R^{N},u_{i}\geq 0,\;i=1,\cdots ,N\right\}. \end{array}$$

This set of equations will define a dynamics on a structural graph with *N* nodes, taking one equation for the description of the dynamics on each of the nodes. We model the dynamics on a given mechanism from Lotka-Volterra systems since they lead to a non-linear and nontrivial class of examples where the characterization of ISs and its dependence on parameters is, to some extent, very well understood [50, 52]. Indeed, we can find, under some conditions on parameters, that there exits a finite number of equilibria (then, with trivial recurrent behaviour) and directed connections between them, generating an hierarchical organization by level sets of equilibria ordered by connections in a gradient-like fashion [19, 20]. With respect to IIT, each IS is associated to a mechanism in a state and gives it intrinsic information.

As noted, we also want each Informational Structure to flow in time. Thus, we can consider the system of differential equations driven by time dependent sources *α*(*t*) (and/or *γ*_*ij*_(*t*)), given by:

$$\begin{array}{c} {\dot{u}}_{i}=u_{i}(\alpha_{i}\left(t\right)+\sum_{j=1}^{N}\gamma_{ij}u_{j}),\;\;\;\;i=1,...,N. \end{array}$$ (6)

#### Informational fields

**Fig 3:**
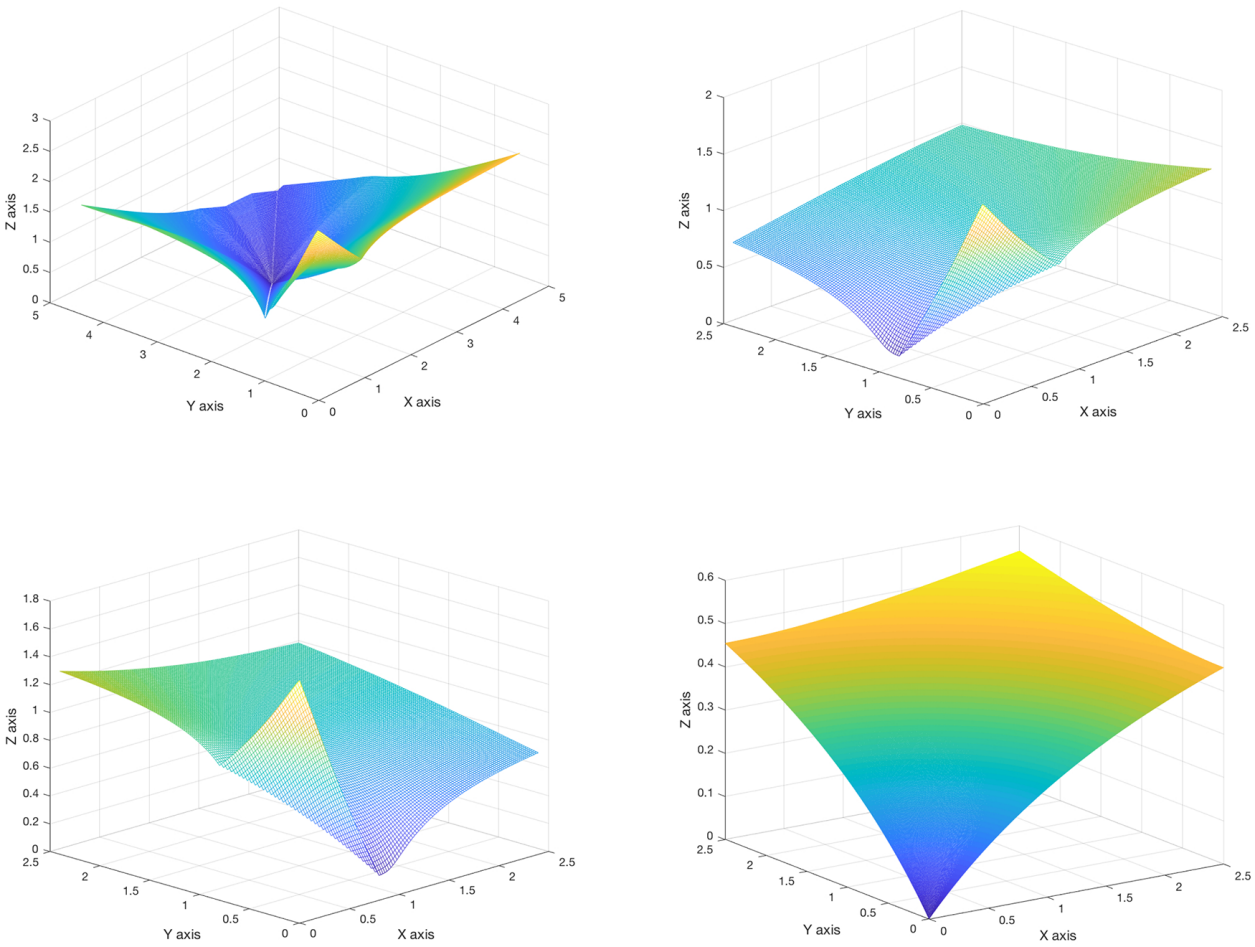
Informational fields. Informational structures are the skeleton of informational fields, the ones with the power of causality to the past and the future in the IS. For a mechanism of two nodes as in Fig 1 there exist different possible ISs. We plot the four ones related to the existence of one, three or four equilibria for equations in (7), which depends on the values of the *α* and *γ* parameters. Observe that the IF produces a curvature of the *XY*-phase space, and governs the dynamics of all the states in the IF. **Top left**. Informational field for the informational structure in Top right in Fig 1, the one for which the four equilibria exist. Note that (0, 0) is unstable, (0, 1) and (1, 0) are saddle equililibria, and (2.5, 3) is a globally stable stationary solution. These stability and instability properties of each node, characterised from their eigenvalues and eigenvectors, define the curvature of the bidimensional plane drawn by the IF. **Top right**. IF for the IS with the three equilibria, the (0, 0) and the semitrivial equilibria (0, 1) in the *Y*-axis and (1, 0) in the *X*-axis, with the global stationary solution in the *Y*-axis. **Bottom left**. IF for the IS with the three equilibria, the (0, 0) and the semitrivial equilibria (0, 1) in the *Y*-axis and (1, 0) in the *X*-axis, with the global stationary solution in the *X*-axis. **Bottom right**. IF where (0, 0) is the only stationary solution, which is globally stable, making a global pending towards this stationary point.

Note that an IS is not only a complex network of possible configurations of a graph. Moreover, it is well organized by their connections, showing the intrinsic dynamics within the IS and, acting as a global attractor, it determines the behaviour of every particular realization in the system. In this sense, it is not only that every point in the IS possesses an amount of information, but every point of the phase space is enriched from the information given by the IS. Indeed, every state of the mechanism is determined by the Informational Field (IF) associated to the IS. See Fig 3 for an illustration of IFs. In this way, the IS can be seen just as the skeleton of a real body organized as a global vector field describing all the possible flows of information in the system. This is why, related to integrated information, it is the IS and its associated dynamics (IF) the object to be studied. Actually, it is the IF what is really responsible of the cause-effect power of a IS.

#### Dynamical informational structures

**Fig 4:**
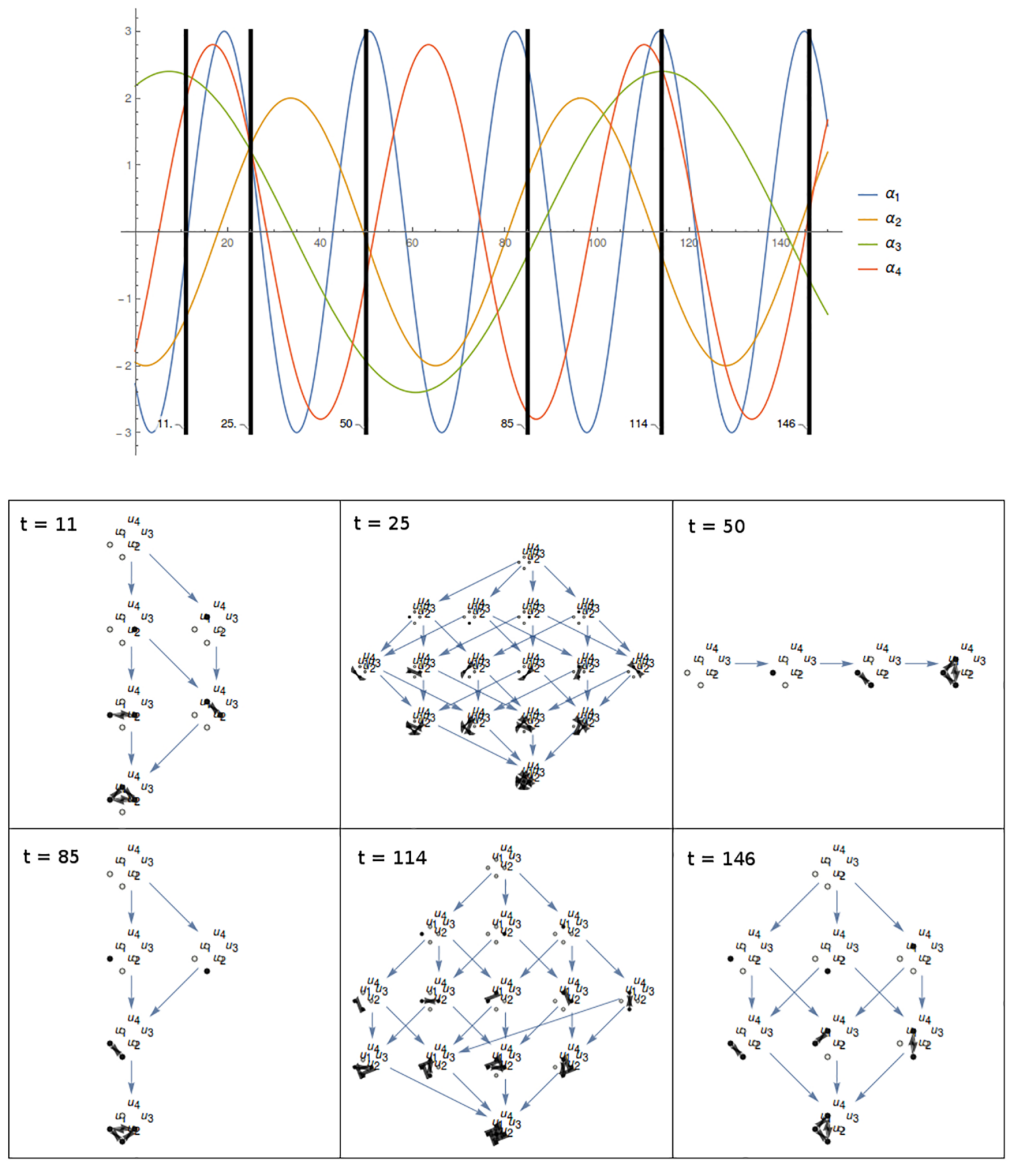
Dynamic informational structures. Changes in the parameters governing the dynamics of a mechanism produce changes in the corresponding informational structures. Actually, these changes can be proved to be continuous with respect to time when, for instance, they are associated to the global model of differential equations in (4). **Top**. Example for the evolution in time of *α*_*i*_ parameters of a system of four nodes; *γ* parameters are fixed. Black bars select six different time points. **Bottom**. For a given mechanism, different ISs corresponding to the time steps shown above.

It seems clear that brain dynamics does not behave by converging or stabilizing around a fix set of invariants, but it can be described as a continuous flow of quick and irregular oscillations [53, 54]. We deal with this situation by introducing dynamic informational structures (DIS), i.e., a continuous time map on informational structures so that their influence can be realized at each time value (Fig 4). We can make parameters in (4) depend on time, so that for each time $t\in R^{+}$ we have an associated Informational Structure $I_{t}$. Thus, we can define Dynamical Informational Structures (DIS) as a continuous flow

$$\begin{array}{c} S:R^{+}\to 2^{I}\\ S\left(t\right)=I_{t},\;\text{for}\;\text{all}\;t\in R^{+} \end{array}$$

which is giving a dynamical behaviour on the complex network of connected subgraphs given by the ISs at each time *t*. $2^{I}$ is the set of subsets of I, where I is the set of all stationary solutions. Note that S induces a continuous movement of structures. The determination of critical values in which topological and/or geometrical changes are crucial, and will be related to bifurcation phenomena of attractors [55, 56].

The reader wishing to retrace our formulation will find all code developed for the implementation of the results and the generation of the technical figures herein from the Open Science Framework (https://osf.io/5tajz/).

## Results

### Integrated information: A continuous approach

In this section we define a preliminary version of IIT postulates: existence, composition, information, integration and exclusion. We do not try to mimic or generalize the concepts of IIT 3.0 (see Section Discussion), where they appear in a more elaborated fashion, allowing finer computations and insights. For example, IIT considers the integrated information of each subset of a mechanism (candidate set), while we always focus on the whole mechanism.

#### Existence: Mechanisms in a state

**Fig 5:**
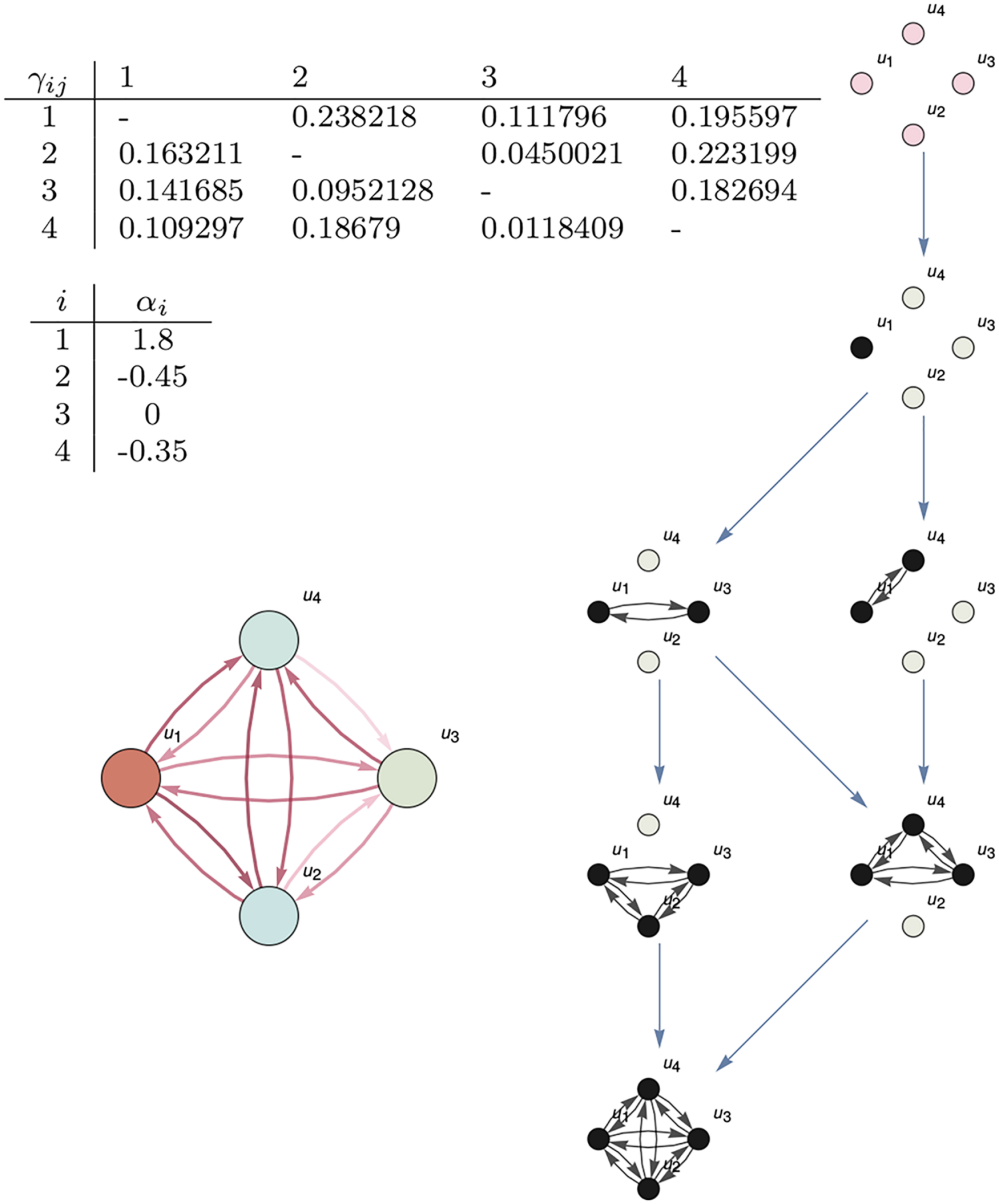
Existence. **Left**: The mechanism is given by a set of nodes (in the figure, *u*_1_, …, *u*_4_) and the *α* and *γ* parameters. Parameters *α*_*i*_ affect only the dynamics of the node *u*_*i*_. Parameters *γ*_*ij*_ model the influence of *u*_*i*_ over *u*_*j*_. **Right**: The dynamics given by (7) on the mechanism generates an informational structure with nodes the non-negative solutions of the corresponding system of differential equations. This structure controls the behaviour of the system (all possible states of the mechanism). There exists a hierarchy (energy levels, represented by horizontal rows) between the points in the informational structure, with just one global stable point and arcs between them, representing solutions going from one point to another. There is an arc from stationary solution $u_{i}^{*}\in R^{4}$ to another one $u_{j}^{*}\in R^{4}$ when the set of non-zero values in $u_{i}^{*}$ is a subset of non-zero values in $u_{j}^{*}$. For clarity, we have removed arcs going to each node to itself and all arcs that can be obtained by transitive closure of those already represented.

We consider mechanisms like in Fig 5. The mechanism contains a set of nodes with links representing the influences between them. On nodes we define a dynamical system driven by the set of differential equations (a particular case of (4)):

ui′(t)=αiui(t)-ui(t)2+∑1≤j≤Nj≠iγijui(t)uj(t) (7)

for each node *u*_*i*_ from 1 to *N* (in Fig 5, *N* = 4). Values *α*_*i*_ are relative to each node. Values *γ*_*ij*_ represent the influence of node *u*_*i*_ over *u*_*j*_.

The dynamics of system (7) generates an attractor, which is a structured set of stationary points (or equilibria) for the system, for which there exists a globally stable stationary point (represented at the right part of Fig 5).

Colours in the nodes of Fig 5 (bottom left) go from dark blue (−3) to red (3) and represent *α*_*i*_ values. Fig 5 (right) shows the whole attractor corresponding to the system on the left with the given *α* and *γ* parameters. We call this attractor the informational structure (IS) for the mechanism. Note that the same mechanism with different *α*_*i*_ and *γ*_*ij*_ values generates different ISs. Nodes in the IS correspond to distinguished states of the mechanism. We call them *states of the IS*. Each of these states is a subgraph of the mechanism in Fig 5 (bottom left) corresponding to a non-negative solution of (7). Black points represent non-zero values in the stationary solutions (i.e., *u*_*i*_ > 0 in the equilibrium) and grey point are zero values (*u*_*i*_ = 0). Each state of the IS governs the behaviour of a family of states of the mechanism, those with the same positive nodes. That is, the cause-effect power of each node of the IS determines the cause-effect power of the mechanism in a state represented by that node.

#### Cause-effect power of a mechanism in a state

The cause-effect power of nodes (i.e., stationary points or, equivalently, states) in an informational structure is represented by defining transition probability matrices (TPMs). Given that the attractor captures the behaviour of all solutions looking to the future (*t* → + ∞) and to the past (*t* → −∞), for each state we introduce two matrices, one with the transitions to the future and the other to the past. The procedure is analogous for both distributions. Observe that we do an approach by probability distributions of both the informational structure and the informational field.

We know that local dynamics on each node (stationary solution) in the IS can be described by the associated eigenvalues and eigenvectors when we linearize (7) on nodes [57]. Indeed, given $f:R^{N}\to R^{N}$ by

fi(u1,⋯,uN)=αiui-ui2+∑1≤j≤Nj≠iγijuiuj,i=1,⋯,N,

the associated *N* × *N* Jacobian matrix is given by $J={\left(J_{ij}\right)}_{1\leq i,j\leq N}\in R^{N^{2}}$, with

$$\begin{array}{c} J_{ij}\left(u\right)=\frac{\partial f_{i}}{\partial u_{j}}\left(u\right),\;1\leq i,j\leq N. \end{array}$$

On each node (state of the IS) $u^{*}\in R^{N}$, we calculate the eigenvalues λ_*i*_ and associated eigenvectors, normalise to one, $w^{i}=\left(w_{1}^{i},\cdots ,w_{N}^{i}\right)\in R^{N}$ for matrix *J*(u*). The exponential of negative eigenvalues gives the the strength of attraction towards the node. The exponentials of positive eigenvalues give the the strength of repulsion from the node, and the repulsion directions are given by the associated eigenvectors of this node. To take into account the influence of the *w*^*i*^, we also take *v^i^* = abs(*w^i^*), the absolute value of *w*^*i*^. Thus, if u* is a saddle node with associated eigenvalues λ_*i*_ = λ_*i*_(u*) > 0, we calculate the *N*-dimensional vector

$$\begin{array}{c} \vec{\text{Rp}}\left(u^{*}\right)=\sum_{\lambda_{i}>0}\lambda_{i}v^{i}, \end{array}$$ (8)

and we define the repulsion rate from u* as

$$\begin{array}{c} \text{Rp}\left(u^{*}\right)=\text{exp}\left(\sum_{1\leq k\leq N}\vec{\text{Rp}}\left(u^{*}\right)\left(k\right)\right). \end{array}$$ (9)

In the same way, if u* is a saddle node with eigenvalues λ_*i*_ = λ_*i*_(u*) < 0,

$$\begin{array}{c} \vec{\text{Att}}\left(u^{*}\right)=\sum_{\lambda_{i}<0}-\lambda_{i}v^{i}, \end{array}$$ (10)

and so the attraction rate to u* is given by the exponential of the sum of the *N*-components of vector Att(u*), i.e.

$$\begin{array}{c} \text{Att}\left(u^{*}\right)=\text{exp}\left(\sum_{1\leq k\leq N}\vec{\text{Att}}\left(u^{*}\right)\left(k\right)\right). \end{array}$$ (11)

**Fig 6:**
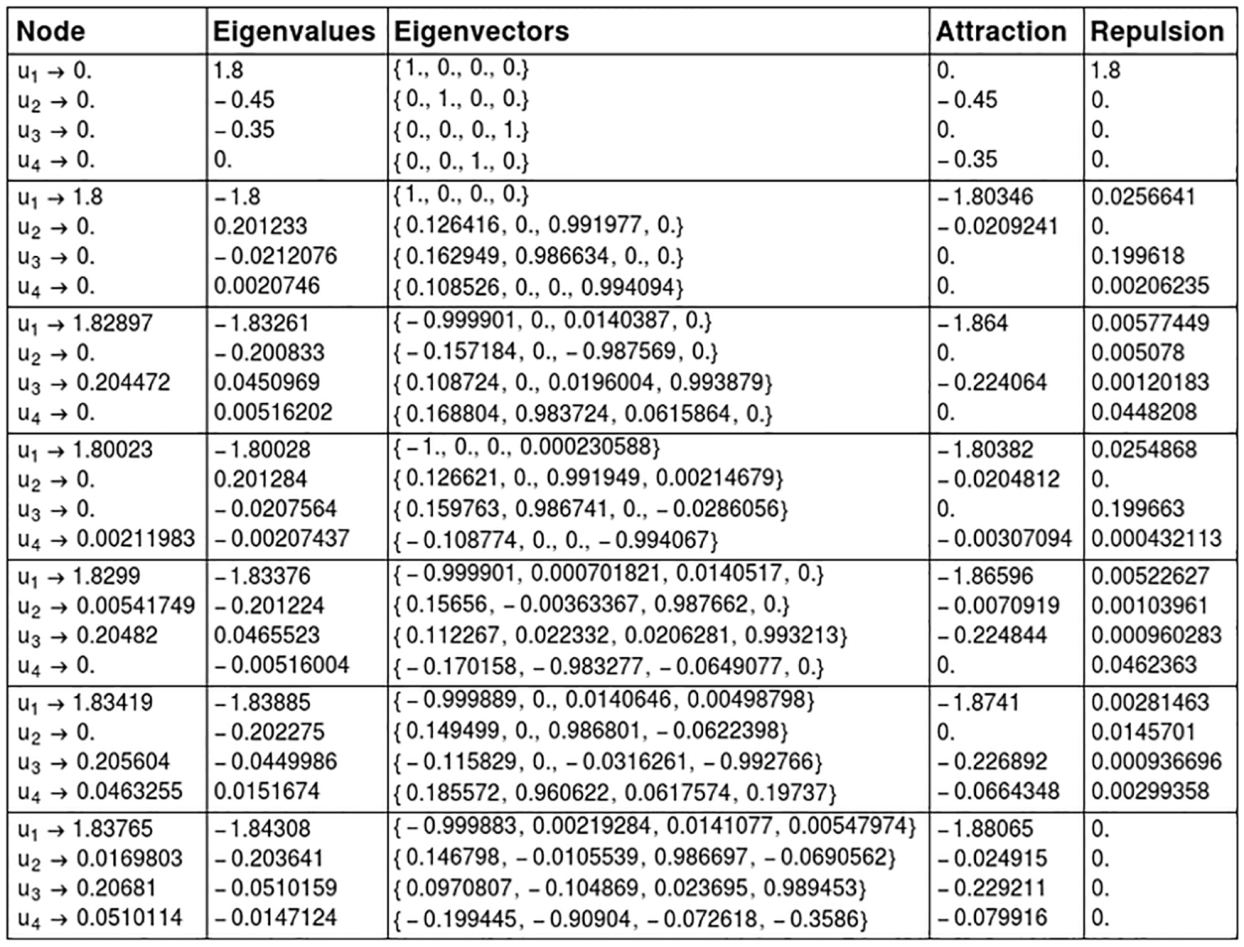
Calculation of the eigenvalues and eigenvectors associated to each stationary solution for the system in Fig 5. Rows correspond to the nodes (states) of the IS. States will be denoted by the list of components with a value greater than 0. This way, the second state, (1.8, 0, 0, 0), will be denoted by (*u*_1_). State (0, 0, 0, 0) is abbreviated by (0). Attraction is calculated from negative eigenvalues by (10), given a vector in $R^{4}.$ Repulsion is calculated by positive eigenvalues from (8), again a vector in $R^{4}$.

Heuristically, this functional on positive eigenvalues reflects the exponential divergence of trajectories in the unstable manifolds being pushed out from a neighbourhood of its associated node; the functional on negative eigenvalues of a node describes the exponential convergence of trajectories in the stable manifold being attracted to the node. Thereby, we reflect the degree to which the saddle fixed points (nodes) locally attracts, or repels trajectories to create a chain with the other nodes in the global attractor. Fig 6 shows and example for this calculation on $R^{4}$.

Our aim is to give a mathematical measure on connections between IS-nodes (i.e., between vertices in the IS). We go through the informational field, which is really causing the deformation of the phase space leading to these connections. Note that we will just consider the strengths which are directly pushing to create a link between nodes, given by the repulsion rates around all the intermediate nodes and the attraction rate to the final node. This is not the only option we could have been considered, as we could also have taken into account the attraction rates between intermediate nodes. This fact would not produce any relevant difference to our treatment. Specifically, if we start at IS-node $u_{i}^{*}$ which is connected in the future to $u_{j}^{*}$, with *i* < *j*, we may pass through the local dynamical action of all intermediate nodes $u_{i+1}^{*},\ldots u_{j-1}^{*}$ between them. In this way, to measure the strength connection (to the future) (S_fut_) from $u_{i}^{*}$ to $u_{j}^{*}$ we sum the repulsion action of $u_{i}^{*}$ given by (9), the (exponential) repulsion from all intermediate nodes. Finally, we add the (exponential) attraction from $u_{j}^{*}$ given by (11). Thus, the strength of the connection (to the future) from $u_{i}^{*}$ and $u_{j}^{*}$ can be written as

$$\begin{array}{c} S_{\text{fut}}\left(u_{i}^{*},u_{j}^{*}\right)=c\sum_{k=0}^{j-i-1}\text{Rp}\left(u_{i+k}^{*}\right)+\text{Att}\left(u_{j}^{*}\right), \end{array}$$

where constant c is a corrector term for the difference between the dimension of the unstable manifold of $u_{i}^{*}$ and the stable manifold of $u_{j}^{*}$, and is given by

$$\begin{array}{c} c=2^{\text{dim}\left(W^{u}\left(u_{i}^{*}\right)\right)-\text{dim}\left(W^{s}\left(u_{j}^{*}\right)\right)}. \end{array}$$

To measure connections in the past we follow a similar argument, by changing the signs of attraction and repulsion values: if we start at IS-node $u_{j}^{*}$ which is connected in the past to $u_{i}^{*}$, with *j* > *i*, we pass through the local dynamical action of all intermediate IS-nodes $u_{j-1}^{*},\ldots u_{i+1}^{+}$ between them. In this way, to measure the strength connection (to the past) from $u_{j}^{*}$ to $u_{i}^{*},$ once we have changed signs, we sum the (exponential) attraction of $u_{j}^{*}$, the (exponential) attraction from all intermediate nodes and finally the (exponential) repulsion dynamics from $u_{i}^{*}$.

The normalization of these values so that their sum is 1 allows a natural translation into probability distributions associated to each state, one for the past and another for the future. Thus, to build the TPM for the future, noted as TPM_fut_ with entries TPM_fut_(*i*, *j*) we make

$$\begin{array}{c} p^{f}\left(u_{i}^{*},u_{j}^{*}\right)=\frac{S_{\text{fut}}\left(u_{i}^{*},u_{j}^{*}\right)}{\sum_{k\geq i}S_{\text{fut}}\left(u_{i}^{*},u_{k}^{*}\right)}, \end{array}$$

with $p^{f}\left(u_{i}^{*},u_{j}^{*}\right)={\text{TPM}}_{\text{fut}}\left(i,j\right)$.

**Fig 7:**
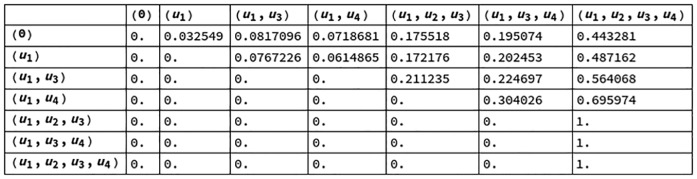
Transition probability matrix for the future of the IS in Fig 5.

**Fig 8:**
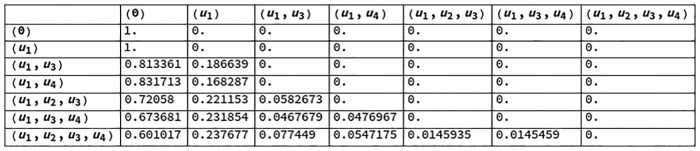
Transition probability matrix for the past of the IS in Fig 5.

Figs 7 and 8 contain the transition probability matrices for the informational structure of Fig 5 (right) by looking, respectively, at the future and the past. States (nodes) in the informational structure are represented (with parentheses as notation) by the list of elements (nodes of the mechanism, Fig 5 left) with a value greater than 0. The state with no elements greater than 0 is represented by (0). Recall that a stationary solution $u_{j}^{*}$ can be reached from $u_{i}^{*}$ if and only if $u_{i}^{*}\subseteq u_{j}^{*}$, in the sense that solutions are represented by the set of nodes with values greater than 0.

#### Information

Inspired by IIT, the level of information of a mechanism in a state is compared both for the past (cause information) and the future (effect information).

In order to introduce the formal definitions, consider an informational structure *IS* with *N* nodes $\left\{u_{1}^{*},\ldots ,u_{N}^{*}\right\}$. Also,

$$\begin{array}{ccc} {\#}_{u_{i}^{*}}^{*} & = & \left|\{u_{j}^{*}|1\leq j\leq N,\;u_{i}^{*}\subseteq u_{j}^{*}\}\right|\\ {\hat{\#}}_{u_{i}^{*}}^{*} & = & \left|\{u_{j}^{*}|1\leq j\leq N,\;u_{j}^{*}\subseteq u_{i}^{*}\}\right| \end{array}$$

${\#}_{u_{i}^{*}}^{*}$ is the number of points in the informational structure accessible from $u_{i}^{*}$ to the future (supersets of $u_{i}^{*}$) and ${\hat{\#}}_{u_{i}^{*}}^{*}$ the number of accessible points from $u_{i}^{*}$ to the past (subsets of $u_{i}^{*}$). Note that each node in the IS determines a subset of it.

#### Cause information

Cause information measures the different probability distributions to the past obtained by considering the knowledge of just the structure (unconstrained past, p^*up*^) and the structure in the current state (cause repertoire, p^*cr*^). The probability distribution for *unconstrained past*, p^*up*^, is obtained by considering that any node $u_{j}^{*}$ can be the actual one with the same probability. Formally,

$$\begin{array}{c} p^{up}\left(u_{i}^{*}\right)=\frac{1}{N}\sum_{j=1}^{N}p^{p}\left(u_{i}^{*}|u_{j}^{*}\right) \end{array}$$ (12)

where *cause repertoire* is directly defined by p^*p*^ in the TPM (past)

$$\begin{array}{c} p^{cr}\left(u_{i}^{*}|u_{j}^{*}\right)=p^{p}\left(u_{i}^{*}|u_{j}^{*}\right), \end{array}$$ (13)

and

$$\begin{array}{c} p^{p}\left(u_{i}^{*},u_{j}^{*}\right)={\text{TPM}}_{\text{past}}\left(i,j\right). \end{array}$$

Cause information is the distance between both probability distributions. Earth mover’s distance EMD (or Wasserstein metric) [58] is used, so that

$$\begin{array}{c} ci\left(IS\right)=EMD\left(p^{up},p^{cr}\right) \end{array}$$ (14)

#### Effect information

Effect information is computed analogously to those presented for cause information. Formally,

$$\begin{array}{ccc} p^{uf}\left(u_{i}^{*}\right) & = & \frac{1}{N}\sum_{j=1}^{N}p^{f}\left(u_{i}^{*}|u_{j}^{*}\right) \end{array}$$ (15)

$$\begin{array}{ccc} p^{er}\left(u_{i}^{*}|u_{j}^{*}\right) & = & p^{f}\left(u_{i}^{*}|u_{j}^{*}\right) \end{array}$$ (16)

$$\begin{array}{ccc} ei(IS) & = & EMD(p^{uf},p^{er}) \end{array}$$ (17)

#### Cause-effect information

Finally, the *cause-effect information* of the informational structure *IS* is the minimum of *cause information* and *effect information*,

$$\begin{array}{c} cei(IS)=min\{ci(IS),\;\;ei(IS)\}. \end{array}$$ (18)

**Fig 9:**
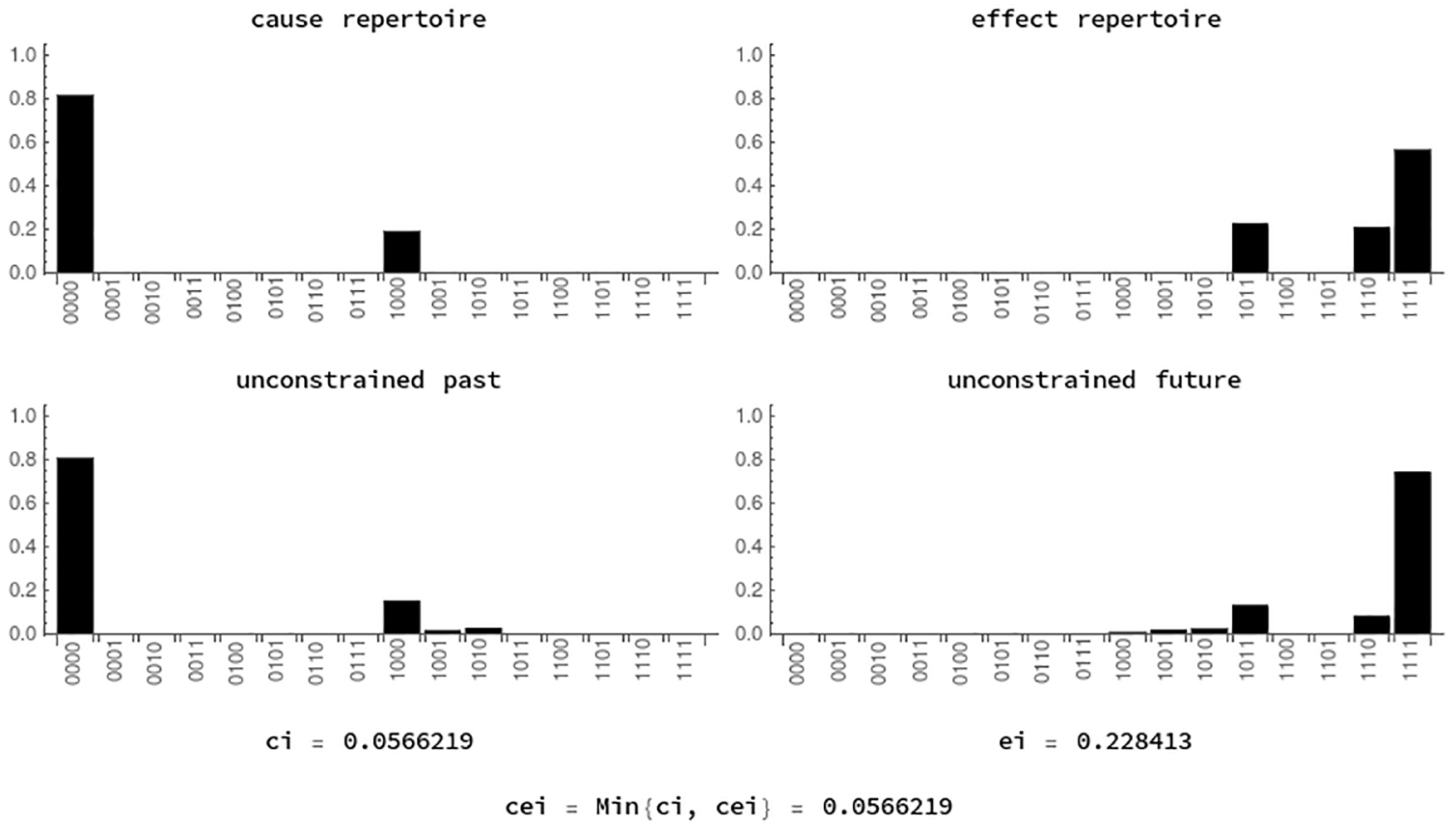
Information in state (*u*_1_, *u*_3_). Cause and effect probabilities of both the structure (unconstrained repertoire given by (12)) and dynamics (cause repertoire from (13)) are compared by using EMD. Cause-effect information (cei) is the minimum of cause information (ci) and effect information (ei).

Fig 9 shows the distributions involved in the calculation of cause-effect information in state (*u*_1_, *u*_3_) of the informational structure in Fig 5 (that is, the state in which only *u*_1_ and *u*_3_ have a value greater than 0).

#### Integration

Information is said to be integrated when it cannot be obtained by looking at the parts of the system but only at the system as a whole. We can measure the integration of an informational structure by trying to reconstruct it from all possible partitions.

As we are considering mechanisms with a dynamical behaviour, when a partition $P/\bar{P}$ of a mechanism with nodes {*u*_1_, …, *u*_*N*_} is considered, the IS is computed by taking apart from the dynamics of each *u*_*i*_ the nodes outside the same partition. Partitions are computed for a mechanism at a given state $u^{*}=\left(u_{1}^{*},\ldots ,u_{N}^{*}\right)$, so in the equation for *u*_*i*_, any node *u*_*j*_ in the other partition is given the constant value $u_{j}^{*}$. So, the IS for the partition $P/\bar{P}$ is not computed by using (7) but

ui′(t)={αiui(t)-ui(t)2+∑uj∈Puj≠uiγijui(t)uj(t)+∑uj∈P¯γijui(t)uj*ifui∈Pαiui(t)-ui(t)2+∑uj∈P¯uj≠uiγijui(t)uj(t)+∑uj∈Pγijui(t)uj*ifui∈P¯ (19)

The informational structure of the partition contains the set of stable points of (19) together with the transition probability matrices for them, as defined above.

**Fig 10:**
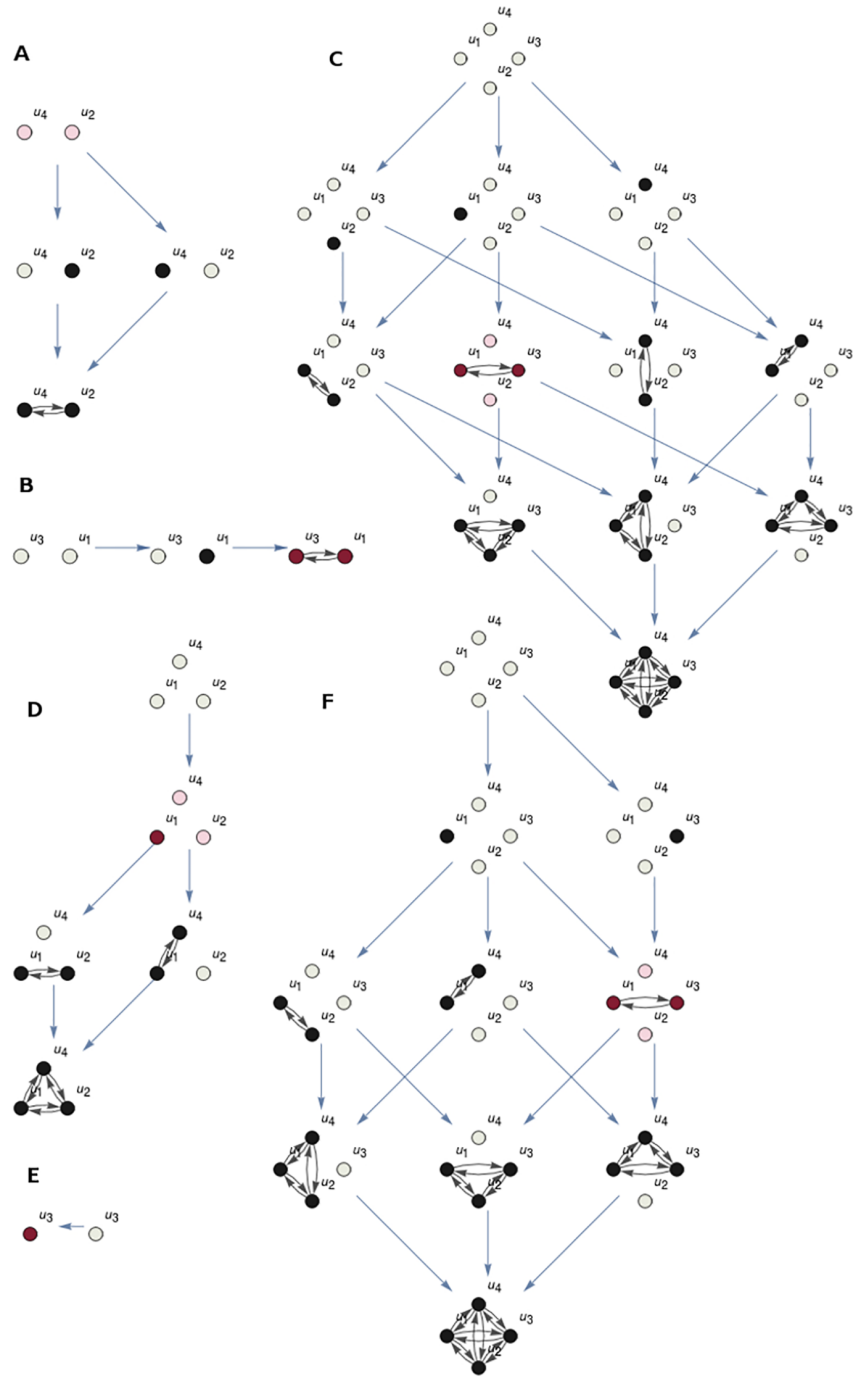
Minimum information partition (MIP) for state (*u*_1_, *u*_3_). (**A**-**C**): Partition {*u*_1_, *u*_3_}/{*u*_2_, *u*_4_} of the system in Fig 5 with the cause repertoire closer to that of the complete system for state (*u*_1_, *u*_3_) (Fig 9). The ISs in **A** and **B** correspond to the submechanisms {*u*_1_, *u*_3_} and {*u*_2_, *u*_4_}, respectively. Nodes in the IS **C** are those in the Cartesian product of **A** and **B**. States of the ISs are highlighted in pink, observe that only *u*_1_ and *u*_3_ have values greater than 0 in the state of each IS, as the partition is for state (*u*_1_, *u*_3_). (**D**-**F**): Partition {*u*_1_, *u*_2_, *u*_4_}/{*u*_3_} with the effect repertoire closer to that of the whole system for state (*u*_1_, *u*_3_).

To measure integration, all partitions with non empty **P** and $\bar{P}$ are considered. Cause and effect repertoires are calculated for all of them, following (12) and (15). The partition with the cause repertoire closer to the cause repertoire of the *IS* is *MIP*^*cause*^, the *minimum information partition* with respect to the cause. The partition with the effect repertoire closer to that of *IS* is *MIP*^*effect*^. Fig 10 shows the minimum information partitions for the informational structure of Fig 5. The *MIP*^*cause*^ is {*u*_1_, *u*_3_}/{*u*_2_, *u*_4_} (top) and *MIP*^*effect*^ is {*u*_1_, *u*_2_, *u*_4_}/{*u*_3_} (bottom).

**Fig 11:**
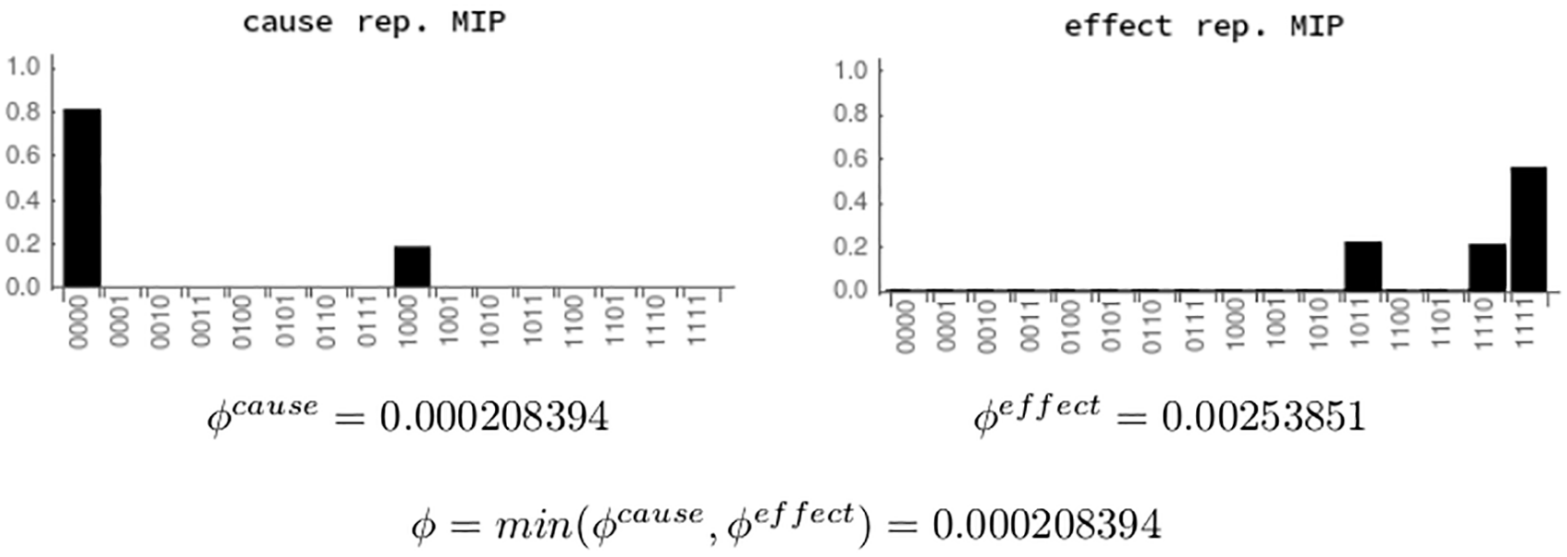
Calculation of *ϕ* for state (*u*_1_, *u*_3_). Cause and effect repertoires of the MIP (partitions for past and future) are compared to the repertoires of the informational structure of the complete system. The integration of the system is given by *ϕ*, the minimum of *ϕ*^*cause*^ and *ϕ*^*effect*^. **Left**: The cause repertoire of the MIP^cause^ (Fig 10, top) at state (*u*_1_, *u*_3_). It is compared by using EMD with the cause repertoire of the whole system (Fig 9, top left) resulting *ϕ*^*cause*^ = 0.000208394. **Right**: *ϕ*^*effect*^ is calculated in an analogous way.

Finally, integration *ϕ* is given by the minimum of *ϕ*^*cause*^ and *ϕ*^*effect*^, where *ϕ*^*cause*^ is the distance between the cause repertoires of *IS* and *MIP*^*cause*^, and *ϕ*^*effect*^ is the distance between the effect repertoires of *IS* and *MIP*^*effect*^. Both distances are calculated using EMD. Fig 11 shows the calculation of *ϕ* for the system of Fig 5 in state (*u*_1_, *u*_3_).

#### Exclusion

Consciousness flows at a particular spatio-temporal grain, and this is the base for the exclusion postulate in IIT 3.0 [21], which we do not develop here (see Section Discussion). First, in a mechanism with many nodes, it may happen that not all of them are simultaneously contributing to the conscious experience. While *ϕ* = 0 for the whole mechanism, it may happen that for some submechanism *ϕ* > 0. Indeed, it may be the case that several submechanisms of the same given mechanism are simultaneously integrating information at a given state. Moreover, consciousness flows in time. But the way it evolves is slower than neuronal firing. In our conception, integrated information is related to the values given by *α* and *γ* parameters in (7). Those parameters may be associated with intensity on connectivity (*γ*_*ij*_) and neuromodulators (*α*_*i*_) which change at a slower flow than neuronal firing [21]. In our approach, small changes in the parameters do not always imply a change in the informational structure, if parameters move within the same *cone* (see [59, 60]). This fact might explain how a conscious experience may persist while neural activity is (chaotically) changing. However, when change moves the parameters to a different cone the IS suffers a bifurcation on its structure, so changing the level of integrated information.

### Topology of a mechanism and integrated information

Although ISs and mechanisms possess quantitative and qualitative major differences on the structure and topology of both networks, it is clear that ISs possess an strongly dependence of their mechanisms’ topology. To show this dependence (but no determination) between mechanisms and associated ISs, we have tried to model the continuous evolution of integrated information for simplified mechanisms. This is probably one of the virtues of our continuous approach to integrated information. In particular, we consider the cases of totally disconnected mechanisms, lattice ones, the presence of a hub, and totally connected mechanisms, showing the key functions of the topology and strength of connections with respect to integrated information. To allow the comparison of the different topologies, the reference value for *α*_*i*_ is 1.6 and for *γ*_*ij*_ is 0.1. The behaviour of *ϕ*-cause and *ϕ*-effect when some of these parameters change is shown for the different mechanisms.

#### Totally disconnected mechanisms

The way we define the cause-effect power of a disconnected mechanism always leads to null integration for the level of information. We highlight that integrated information is positive only if we connect the parts in the mechanism, also showing the dependence on the value of integrated information related to the continuous change on the strenght of the connecting parameter.

**Fig 12:**
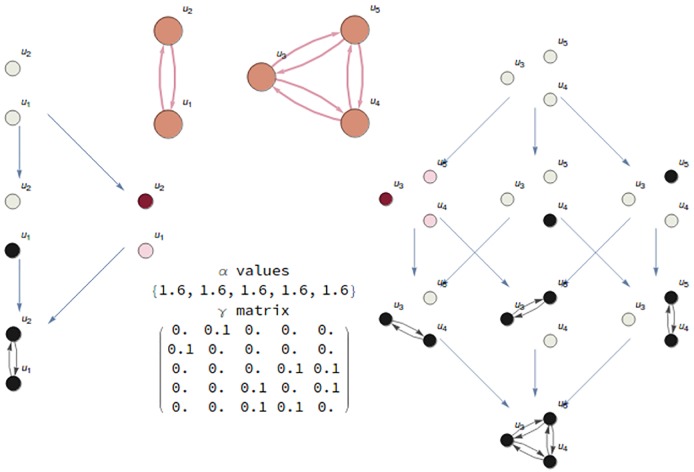
A totally disconnected mechanism. **Top**: Disconnected mechanism. **Left and right**: Each of the partitions in the disconnected mechanism has an associated IS. The nodes of the IS of the whole mechanism (not represented in the figure) correspond to the Cartesian product of both smaller ISs. For example, the values for nodes *u*_2_ and *u*_3_ in the state (*u*_2_, *u*_3_) of the whole IS are the same that their values at states (*u*_2_) and (*u*_3_) of the left and right ISs, respectively (states highlighted in pink). **Bottom**: *α*_*i*_ and *γ*_*ij*_ values for the mechanism.

Fig 12 shows an example of a disconnected mechanism. There are two groups of nodes {*u*_1_, *u*_2_} and {*u*_3_, *u*_4_, *u*_5_}. Connections are only inside each group but not from one group to the other. The consequence is that the IS of the mechanism behave like the Cartesian product of the ISs of the partition (left and right ISs in the figure).

**Fig 13:**
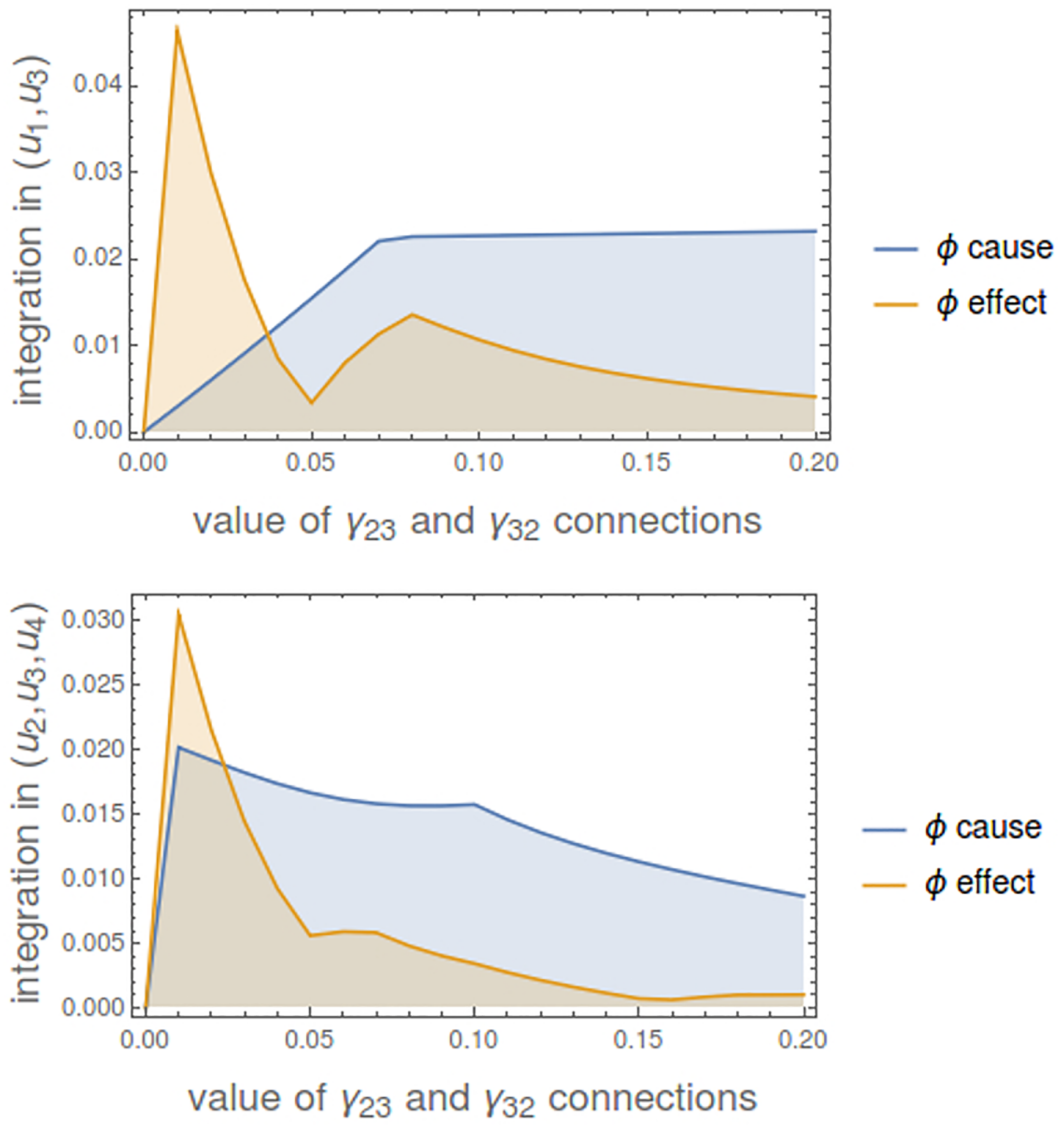
Integration of the mechanism in Fig 12 as we increase the value of the connections between *u*_2_ and *u*_3_. Values of *ϕ*-cause and *ϕ*-effect are shown. The resulting *ϕ* = *min*(*ϕ*^*cause*^, *ϕ*^*effect*^) at each point is the minimum of both values. **Top**: Integration in state (*u*_1_, *u*_3_). **Bottom**: Integration in state (*u*_2_, *u*_3_, *u*_4_).

We can set the connections between *u*_2_ and *u*_3_, looking at the integration in different states of the mechanism (Fig 13). As expected, when *γ*_23_ = *γ*_32_ = 0 there is no integration.

#### Cyclic mechanisms

**Fig 14:**
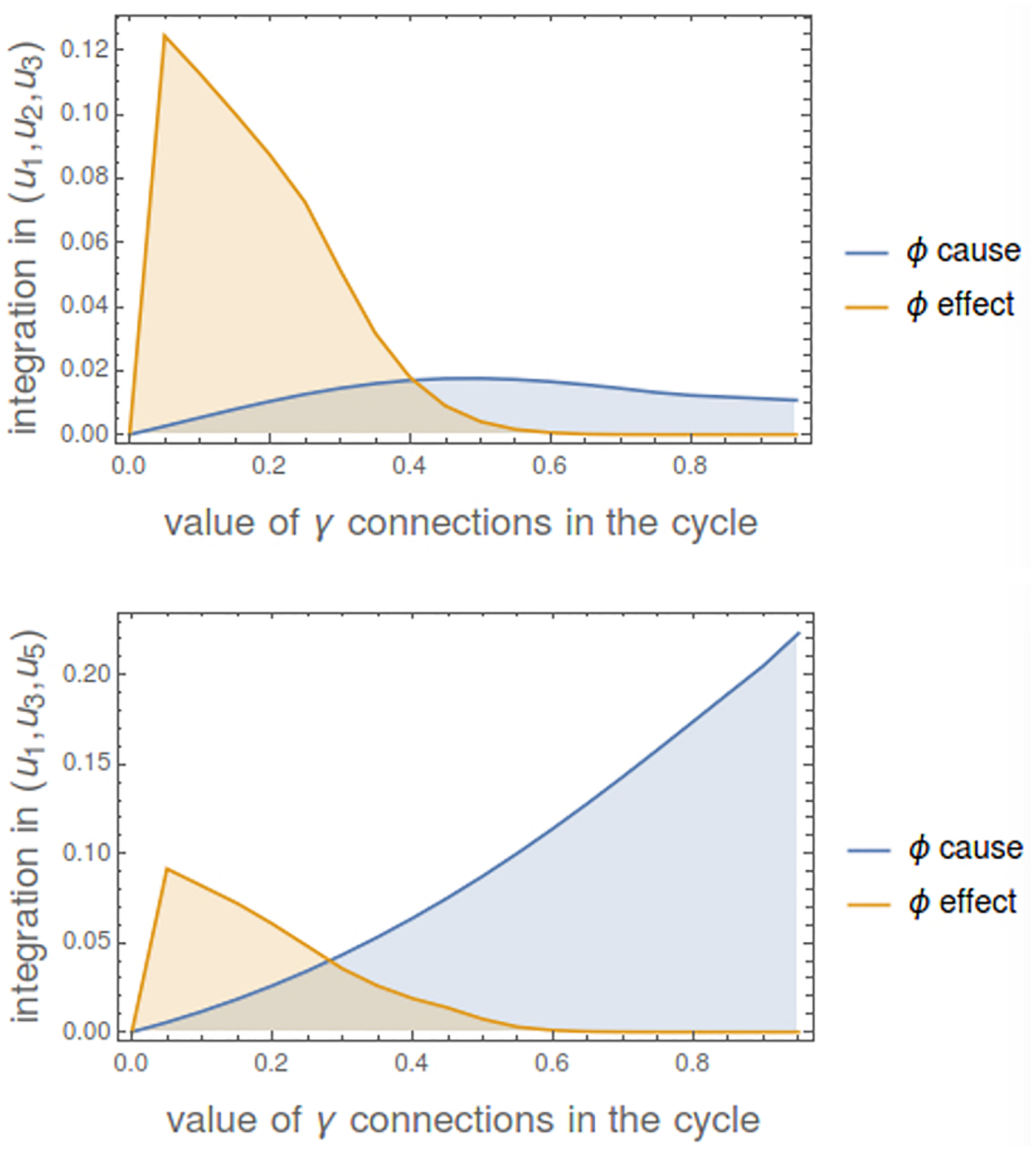
Integration in a cyclic mechanism. **Top**: Integration in state (*u*_1_, *u*_2_, *u*_3_). **Bottom**: Integration in state (*u*_1_, *u*_3_, *u*_5_). Note that the level of integrated information is not only a consequence of the topology of the mechanism (a lattice one in this case), but also on the strength of the connecting parameters and the particular state. In case of state (*u*_1_, *u*_2_, *u*_3_) the three nodes with a positive value are consecutive and in (*u*_1_, *u*_3_, *u*_5_) while *u*_1_ and *u*_5_ are linked in the cycle, *u*_3_ is separated.

Now we consider a mechanism of 5 nodes {*u*_1_, *u*_2_, *u*_3_, *u*_4_, *u*_5_} with all *α*_*i*_ values equal to 1.6. Connections *γ*_*ij*_ create a cycle so that all of them are 0 except {*γ*_12_, *γ*_23_, *γ*_34_, *γ*_45_, *γ*_51_} that have the same value. Fig 14 shows the changing level of integration of the mechanism in states (*u*_1_, *u*_2_, *u*_3_) and (*u*_1_, *u*_3_, *u*_5_) as the strength of the connections grows up. If we look at *min*(*ϕ*- cause, *ϕ*- effect), it is 0 when connections are 0, then it grows up to some maximum and then return to 0.

#### Small world mechanisms: Presence of hubs

**Fig 15:**
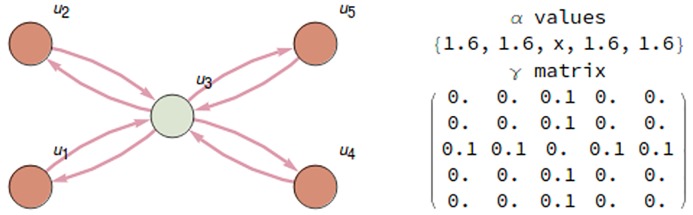
A mechanism with a hub node *u*_3_. Values of parameters *α*_*i*_ and *γ*_*ij*_ are shown at the right, with *α*_3_ = *x* acting as a variable.

**Fig 16:**
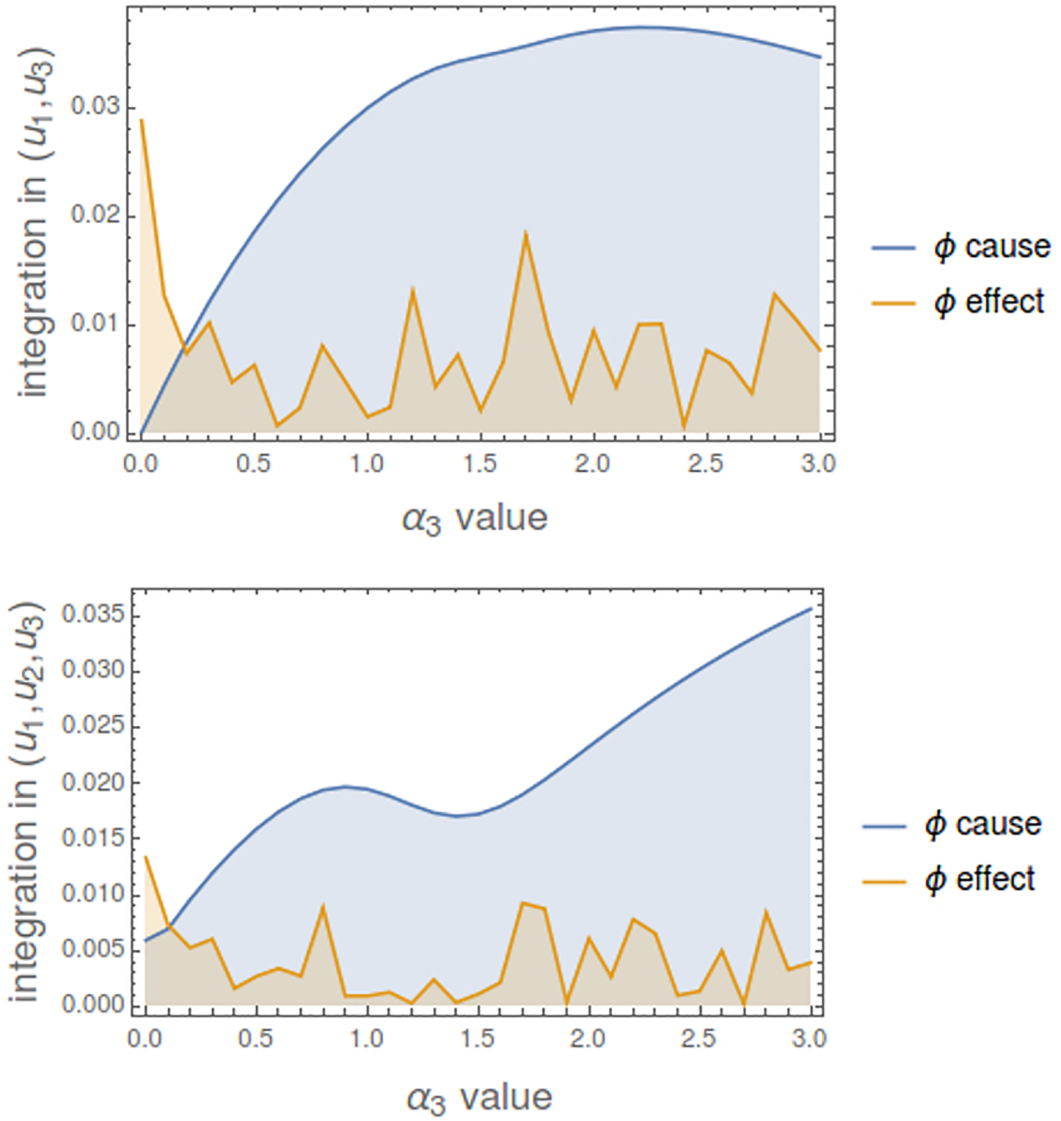
Integration in different states of the hub mechanism of Fig 15 as *α*_3_ increases. **Top**: Integration in state (*u*_1_, *u*_3_). **Bottom**: Integration in state (*u*_1_, *u*_2_, *u*_3_).

Fig 15 shows a mechanism where node *u*_3_ has the role of a hub connecting all other nodes. Fig 16 shows the integration of the system in two different states as the value of *α*_3_ changes. Observe that in this case *ϕ*-effect is more sensible than *ϕ*-cause to small changes on the strength of the hub connection.

#### Totally connected mechanisms

**Fig 17:**
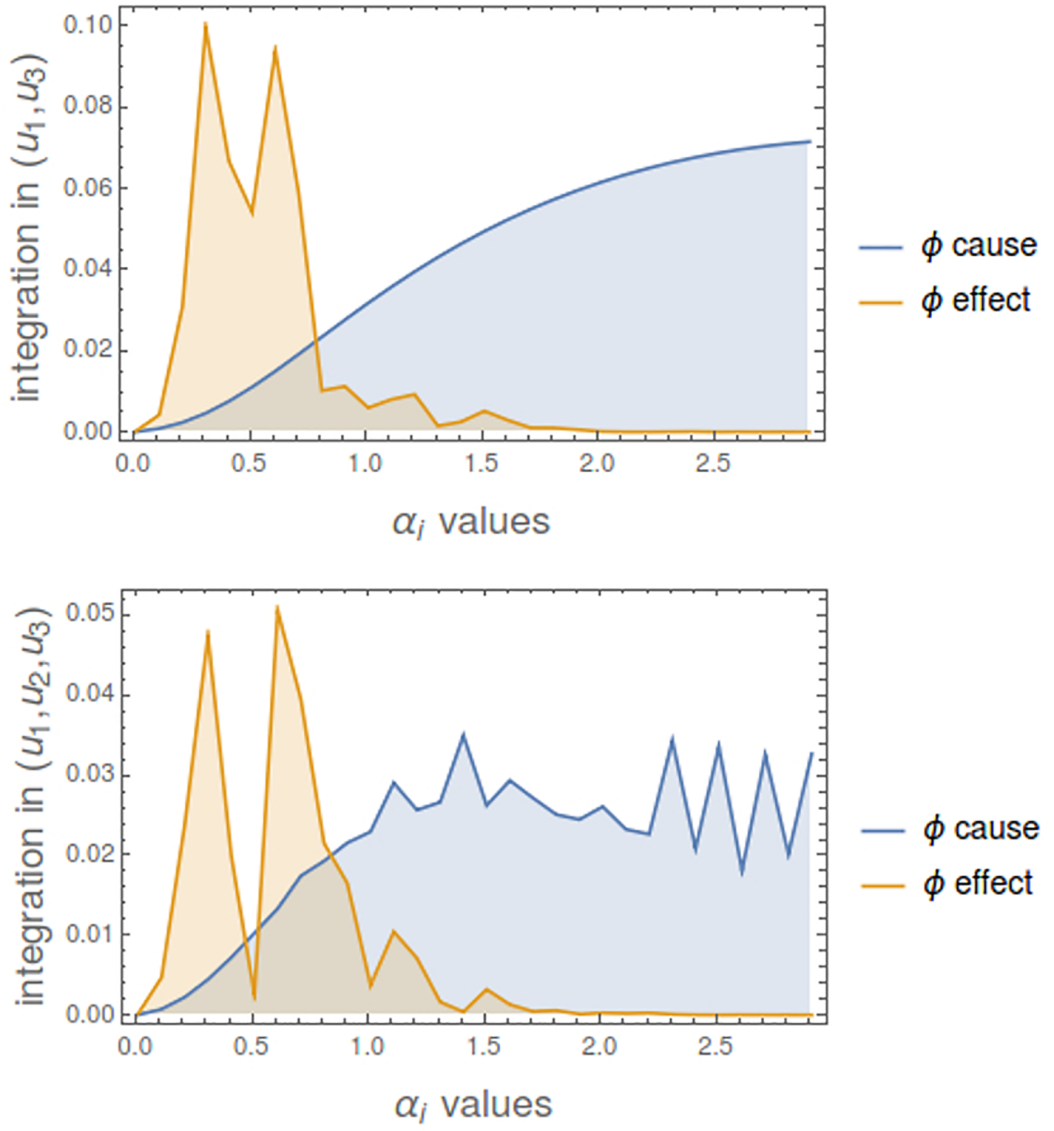
Integration in a totally connected mechanism. Parameters *γ*_*ij*_ are all equal to 0.1. The evolution of *ϕ*-cause and *ϕ*-effect when *α*_*i*_ values go from 0 to 3 is shown for two different states.

We consider now a totally connected mechanism of 5 nodes with all *γ*_*ij*_ values equal to 0.1. Fig 17 shows how integration changes as the *α*_*i*_ values change. It is observed that, even for a totally connected mechanism, integrated information is a delicate measure which generically does not hold with positive values. In both states, when *α*_*i*_ values are greater to 2, integration (*ϕ*-effect) is 0.

## Discussion

### Dynamics on complex networks characterized by informational structures

The concept of informational structure is not only related to the understanding of brain processes and their functionality but, more broadly, as an inescapable tool when analyzing dynamics in complex networks [19, 20]. For instance, in Theoretical Ecology and Economy related to the modeling of mutualistic systems [61, 62], a very important subject is the study of the dependence between the topology of complex networks (lattice systems, unconnected systems, totally connected ones, random ones, “small world” networks) and the observed dynamics on their sets of solutions. The *architecture of biodiversity* [63, 64] is thus referred as the way in which cooperative systems of plants-animals structure their connections in order to get a mechanism (complex graph) achieving optimal levels in robustness and life abundance. The nested organization of this kind of complex networks seems to play a key role for higher biodiversity. However, it is clear that the topology of these networks is not determining all the future dynamics [65], which seems also to be coupled to other inputs as modularity [66] or the strength of parameters [67]. This is also a very important task in Neuroscience [54, 68]. Informational structures associated to phenomena described by dynamics on complex networks contain all the future options for the evolution of the phenomena, as they possess all the information on the forwards global behaviour of every state of the system and the paths to arrive into them. Moreover, the continuous dependence on the parameters measuring the strength of connections and the characterization of the informational structure allow to understand the evolution of the dynamics of the system as a coherent process, whose information is structured, so giving a comprehension to the appearance of sudden bifurcations [69].

From a dynamical system point of view [44], informational structures introduce several conceptual novelties in the standard theory. In particular, for phenomena associated with high dimensional networks or models by partial differential equations [15, 18, 29]. On the one hand, an informational structure has to be understood as a *global attractor at each time*, i.e., we do not reach this compact set as the result of the long time behaviour of solutions, but it exists as a complex set made by selected solutions which is causing a curvature of the whole phase space at each time. Because we allow parameters to move in time, the informational structure also depends on time, so showing a continuous deformation of the phase space by time evolution. It is a real space-time process explaining all the possible forwards and internal backwards dynamics. On the other hand, the understanding of attracting networks as informational objects is also new in this framework, allowing a migration of this subject into Information Theory. It is remarkable that dynamical systems cover from trivial dynamics, as global convergence to a stationary point, to the much more richer one usually referred as chaotic dynamics [43, 44, 57] and dynamics of catastrophes [70]. While the first one can be found with total generality, attractors with chaotic dynamics can be only described in detail in low dimensional systems.

Note that some of the classical concepts of dynamical systems have been reinterpreted. In particular, the global attractor it is understood as a set of selected solutions (organized in invariant sets and their connections in the past and future) which creates the informational structure. This network, moreover, produces a global transformation of the phase space (the informational field) enriching every point in the phase space on the crucial information on possible past states and possible future states. It is the continuous change on time of informational structures and fields what allows to talk on an informational flow for which we can analyse the postulates of IIT. Our approach deals with dynamics on a graph for which a global description of the asymptotic behaviour is posible by means of the existence of a global attractor. That is, dissipative dynamical system for which a global attractor exists. It is important to remark the Fundamental Theorem of Dynamical Systems in [33], inspired in the work of Conley, because it gives a general characterisation of the phase space by gradient and recurrent points, so leading to a global description of the phase space as invariants and connections among them. This is the really crucial point which allows for a general framework on the kind of systems to be considered. The Lotka-Volterra cooperative model we consider is for the applications to IIT, because in this case we have a detailed description of the global attractor, which allows to make concrete computations for the level of integrated information on it. Thus, in application to L-V systems, we are considering heteroclinic channels inside the global attractor. But all the treatment in the paper can be done for every dissipative systems for which a detailed description of the global attractor is available, i.e., this description has not be to be restricted to heteroclinic channels coming from L-V models. Gradient dynamics [15, 27, 29, 31] describes the dynamics from heteroclinic connections and associated Lyapunov functionals [29, 32], and it naturally suits into higher order systems including infinite dimensional phase spaces [27, 34, 35]. Thus, although our description of informational structures associated to Lotka-Volterra systems is general enough to describe real complex networks, we think the concept may be also well adapted both for different topologies in the networks and also different kind of non-linearities defining the differential equations [51, 52]. Actually, for comparison with data and experiments associated for real brain dynamics we certainly need to allow much more complex networks as primary mechanisms as well as different kinds of dynamical rules and nonlinearities. But, in all of them, we expect to find the existence of dynamical informational structures providing the information on the global evolution of the system. The concise geometrical description of these structures and their continuous dependence on sets of parameters is a real open problem in the dynamical systems theory.

### A continuous approach to integrated information

We have presented a preliminary approach to the notion of integrated information from informational structures, leading to a continuous framework for a theory inspired by IIT, in particular in order to define integrated information of a mechanism in a state. The detailed characterization of informational structures comes from strong theorems in dynamical systems theory about the characterization of global attractors. The description of causation in an informational structure by a coherent and continuous flow of integrated information is new in the literature. Indeed, the definition of TPMs between two equilibria in the IS by the strength of eigenvalues and direction of eigenvectors associated to these and intermediate equilibria produces a global analysis of the level of information contained in a particular IS. The same procedure for partitions of the associated mechanism allows a preliminary measure for integration. Moreover, the continuous dependence of the structure of ISs on parameters also opens the door to the analysis to sudden bifurcations, from an intrinsic point of view, for critical values of the parameters. It seems also very clear the close dependence between the topology of a mechanism, the actual value of the parameters and the current state with respect to its level of integrated information, so pointing for optimality for a “small world” configuration of the brain [71].

### Global brain dynamics from informational structures and fields

With respect to global brain dynamics, we think the concept of informational structures and informational fields could serve as the abstract objects to describe functionality and global observed dynamics now described by other concepts and methodologies as multistability [6] or metastability [72, 73]. In [53] the notion of *ghost attractors* is used, which would seem to suit into our informational fields and its bifurcations under the movement of the coupling parameter in the connectivity matrix. We think it could also serve as a valuable tool in line of the perspectives developed in [5]. In [54] a detailed study of different attractor repertoires is studied for a global brain network for which a (local, global and dynamic) Hopfield model is defined. Note that, depending on parameters, the authors observe the bifurcation of dozens or even hundreds of stationary hyperbolic fixed points, which basis of attraction and stability properties has to be analyzed in an heuristic way by simulation of thousands of realizations related to initial conditions. The comparison with empirical data occurs at the edge of multistability. All of these phenomena could naturally enter into a broader global approach, by the study of an abstract formulation of their associated informational fields, creating a network of attractors and their connections, and their dependence on parameters to better understand transitions and bifurcations of structures. Moreover, the continuous approach for ISs possibly leads, if we are able to fit them to real data of brain activity, to a useful tool to analyze the functional connectivity dynamics [74].

### Limitations and future work

The ability to mathematically represent human consciousness can help to understand the nature of conscious processes in human mind, and the dynamical systems approach may indeed be also a correct tool to start this trip towards a complementary mathematical approach to consciousness. Indeed, informational structures allow to associate the processes underlying consciousness to a huge functional and changing set of continuous flow structures. However, we think we are still far to the aim of describing a conscious experience with the actual development, which will be continued in the near future. We have introduced the postulates for IIT associated to ISs, and, in particular, we have developed a first approach for definitions of information and integration, leading to the introduction of measures as *cei* (cause-effect information) or *ϕ* (integrated information of a mechanism).

However, in order to make this approximation comparable to the latest published IIT version for discrete dynamical systems of this theory [21], a series of additional developments are required: In IIT 3.0 the exclusion postulate is applied to the causes and effects of the individual mechanisms, so that only the purview that generates the maximum value of integrated information intrinsically exists as the core cause (or core effect) of the candidate mechanism that generates a *concept*. In fact, a concept is defined as a mechanism in a state that specifies a maximally irreducible cause and effect, the core cause and the core effect, and both specify what the concept is about. The core cause (or effect) can be any set of elements, but only the set that generates the maximum value of integrated information in the past (or future) exists intrinsically as such. We can see the present work as a simplified particular case in which the candidates to core cause and effect, the candidate set to be a concept and the complete system match. But in general they will be different sets. In addition, elements outside the candidate set should be treated as background conditions. On the other hand, the partitions in this work isolate one part of the system from another for any instant of time. However, in line with IIT 3.0, partitions should be performed in this way: two consecutive time steps are considered (past and present or present and future) and the elements of the candidate mechanism in the present are joined to the elements of the candidate to core cause in the past (or core effect in the future) to form a set that will be separated in two, so that in each of the parts there may be elements of the two time steps or not. It is also necessary to distinguish between mechanisms for which the value of *ϕ* for each concept or *quale sensu stricto* is calculated, and systems of mechanisms for which Φ for each complex or *quale sensu lato* is calculated. For the later a conceptual structure or constellation of concepts is defined as the set of all the concepts generated by a system of mechanisms in a state. It is necessary to specify how the conceptual information (CI) and the integrated conceptual information Φ of that system and its corresponding constellation of concepts are calculated. To this aim an extended version of the earth mover’s distance (EMD) is used. When the exclusion postulate is applied to systems of mechanisms, the complexes are defined as maximum irreducible conceptual structures (MICS), that is, local maxima of integrated conceptual information. To do this we should use unidirectional partitions with which to verify that integrated conceptual structures are formed and see if apart from the major complex, and in a non-overlapping way, minor complexes are formed. We are now working in all of these items, and hope, with a much higher computational complexity, a refinement of the results, also in relation to the topology of mechanisms and the integrated information.

Note that the aim to relate (informational) fields and matter by a mathematical treatment is not restricted to our approach or even IIT theory. It is remarkable in this line of research the description of central neuronal systems (and more generically, biological systems) as a gauge theory where a lagrangian approach to model motion is the crucial adopted formalism ([75, 76]). Our techniques are different, since we move into a more classical approach given by dynamical systems related to differential equations. In this way we take advantage of the huge theory on the qualitative analysis of differential equations. In particular, the curvature in the phase space produced by the informational field, closely related to the local stability of stationary points given by their associated eigenvalues, is translated into dynamical properties in the physical space, as a gauge theory approach. But the informational field, in our case, is, for each time, a global transformation of the phase space. This characterisation of an energy landscape (the phase space) evolves continuously in time. It remains an interesting and outstanding challenge to relate gauge theoretic treatments based upon information geometry (for example, the free energy principle) ([76], S3 appendix, or [77]) to our dynamical formulation; however, their common focus on information geometry (and manifold curvature) may admit some convergence in the future. This framework may provide a promising and fruitful perspective.

Thus, our approach provides many open questions and possibilities for future work. Three key problems arise at this stage, showing the necessity for a really interdisciplinary work in the area: the necessity to define the level of information of a global attractor (a totally new question in dynamical systems’ theory), which notion of information better suits to our purposes, and how this concept of information is related to causality [78] on past and future events. We need to continue the development of the dynamical systems version of IIT 3.0, finding the intrinsic representation of cause-effect repertoires and a measure to assess the quantity and quality of integrated information encoded in the informational structure. We also need to represent the evolution in continuous time of this information, and to find a way to measure the strength of attraction and repulsion through measure and dimension of stable and unstable sets and their intersections. Finally, use this notion to define the measure of integrated information compliant with IIT’s Φ^*max*^ and describe the dynamics of this measure, by also creating computer codes for the informational structures evolution.

Informational structures are really grounded in the topology of their associated mechanisms. In a very natural way, we can combine the (continuous) relation between structural networks and informational structures. A huge further research is probably deserved on this subject, not only in neuroscience, but in the huge area of dynamics on complex networks. This approach also opens the door to the development of a theory on the dependence of the structure of informational structures on parameters and on the topology on the complex graphs supporting them. To this aim, we need a theory on bifurcations of invariants and a theory on bifurcation of attractors, both in the autonomous and non-autonomous cases.

In order to the comparison of this approach with real data, we need to develop global models for brain dynamics where the functionality of informational structures and informational fields can be tested. We will need to use the global models of brain dynamics [4, 5, 13, 74, 79], allowing to the use of a brain simulator [80] which correlates with functional networks, and functional connectivity dynamics as developed from real data on human brains.
